# High-throughput production of kilogram-scale nanofibers by Kármán vortex solution blow spinning

**DOI:** 10.1126/sciadv.abn3690

**Published:** 2022-03-16

**Authors:** Ziwei Li, Zhiwen Cui, Lihao Zhao, Naveed Hussain, Yanzhen Zhao, Cheng Yang, Xinyu Jiang, Lei Li, Jianan Song, Baopu Zhang, Zekun Cheng, Hui Wu

**Affiliations:** 1 State Key Laboratory of New Ceramics and Fine Processing, School of Materials Science and Engineering, Tsinghua University, Beijing 100084, China.; 2 Applied Mechanics Laboratory, Department of Engineering Mechanics, Tsinghua University, Beijing 100084, China.

## Abstract

The interaction between gas flow and liquid flow, governed by fluid dynamic principles, is of substantial importance in both fundamental science and practical applications. For instance, a precisely designed gas shearing on liquid solution may lead to efficacious production of advanced nanomaterials. Here, we devised a needleless Kármán vortex solution blow spinning system that uses a roll-to-roll nylon thread to deliver spinning solution, coupled with vertically blowing airflow to draw high-quality nanofibers with large throughput. A wide variety of nanofibers including polymers, carbon, ceramics, and composites with tunable diameters were fabricated at ultrahigh rates. The system can be further upgraded from single thread to multiple parallel threads and to the meshes, boosting the production of nanofibers to kilogram scale without compromising their quality.

## Teaser

A needleless solution blow spinning system based on Kármán vortex is developed for high-throughput production of nanofibers.

## INTRODUCTION

Nanofibers enjoy the luxury of being one of the most attractive nanomaterials because of their unique physicochemical properties and characteristics (*1*–*4*), with numerous existing and ever-expanding practical applications in diverse areas such as environmental filtration, energy conversion and storage, flexible electronics, tissue engineering, thermal insulation, smart textiles, and personal protection against coronavirus disease 2019 (*5*–*14*).To promote the further success of nanofiber materials to user-end products, it is essential to develop more advanced manufacturing science and techniques that can simultaneously provide high quality, high production rate, low cost, easy maintenance, and high reliability to fulfill industrial requirements.

Some nanofiber fabrication methods, including template synthesis, hydrothermal methods, and molecular self-assembly, remain at the laboratory-scale development stage because of complex devices and low production rates (*15*). Melt blowing has proven to be scalable for the commercial production of thin fibers. However, it can hardly produce fibers with diameters less than 1 μm, and only thermoplastic polymers can be produced by using this technique (*16*, *17*). Solution spinning strategies including electrospinning (*18*–*20*), centrifugal spinning (*21*, *22*), mechanical drawing (*23*, *24*), and solution blow spinning (SBS) (*25*, *26*) have received extensive attention as promising routes to fabricate various kinds of ultrathin fibers. In a typical solution spinning process, polymer solution is ejected from a needle tip and stretched by means of electrostatic force (in electrospinning), centrifugal force (in centrifugal spinning), or gas shearing force (in SBS), followed by a subsequent evaporation of solvents to obtain dry fibers. Electrospinning (needle-based or needleless) is a widely adopted solution spinning technique for fabricating nanofibers. However, its major technological downsides, including complicated electric field design, difficulties on solvent evaporation and fiber collection, high-voltage safety concerns, needle obstruction, droplet formation, and most importantly, low yield, have prevented its widespread use in industrial settings. Centrifugal spinning can achieve high fiber productivity, but it still suffers from higher average fiber diameters compared to electrospinning. Therefore, despite rapidly proceeding of existing nanofiber synthesis technologies, manufacturing them at an industrial-scale throughput without compromising their quality standards continues to remain challenging (*15*, *27*–*31*).

High-speed gas flow, as an alternative to electrostatic fields, has recently attracted increasing attention as a driving force to spin ultrathin fibers from solution (*25*, *26*, *29*). In a typical SBS process, a gas flow–induced shearing force generated at the gas-liquid interface is used to refine the solution extruded from a needle tip, forming a liquid jet along the streamwise direction. Subsequently, a gas flow effectively assists in solvent evaporation, leaving behind high-quality dry fibers. To the best of our knowledge, only needle-based SBS techniques have been reported to date, and needleless SBS has not been realized yet (*15*, *29*, *32*). While delivering solutions through needles has achieved much success, problems such as high flow resistance, droplet formation and ejection, and the much likely needle blocking caused by fast solvent evaporation and/or solidification of the solution greatly limit the stability of spinning systems, fiber quality, and production throughput. In addition, it will be quite lucrative for SBS process if the gas shearing stresses with the liquid jet can be further reinforced. Such reinforcement can significantly strengthen the stretching effect and the solvent evaporation during solution spinning. Therefore, it is highly desirable to develop a new SBS system, in which gas flow interacts with the spinning solution in a more efficient way to produce fine fibers with high throughput.

In 1911, Theodore von Kármán (1881–1963) clarified an unusual alternating stream of vortices that resulted from fluid flowing past a cylinder. This ubiquitous phenomenon, now known as a Kármán vortex (KV) street, can be found at all scales of fluid motion (fig. S1) (*33*). It will be of great scientific interest and importance if the gaseous KVs can be explored to manipulate liquid jets and further to facilitate fiber formation. Here, we designed and built a needleless KV-SBS system to realize high-throughput production of nanofibers. We used a rolling nylon thread to continuously deliver spinning solution to a vertically blowing high-speed airflow. The entire solution was ejected as a continuous jet, whipped, and dried to form uniform nanofibers inside the KV street. The gas shearing force and wake turbulence were significantly enhanced by the KV effect, benefitting the stretching and the solvent evaporation of the liquid jets. We demonstrated that this versatile KV-SBS can fabricate a variety of nanofibers at a production rate of up to 5.90 g/hour per jet. The KV-SBS system was further upgraded from single nylon to multiple threads and meshes to produce kilogram-scale nanofibers with high efficiency, demonstrating its suitability for industrial-scale manufacturing.

## RESULTS

### KV-SBS system and working mechanisms

**Fig. 1.**
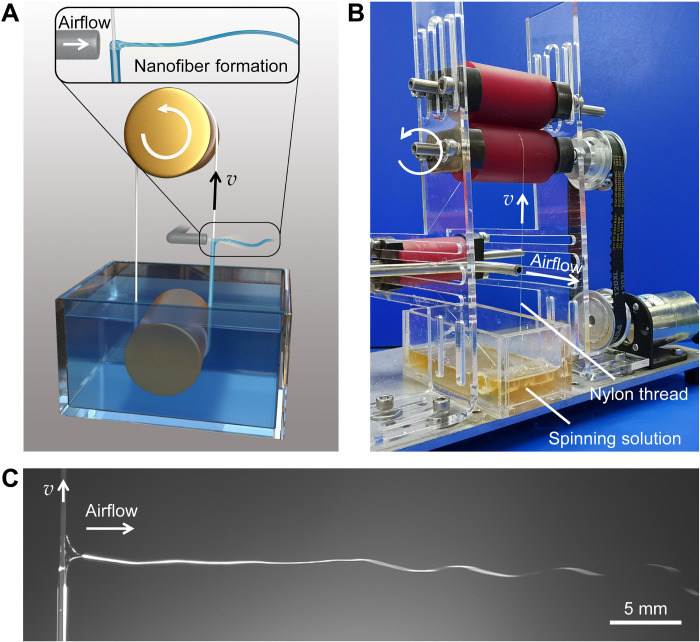
KV-SBS system design and construction. (**A**) Illustration of the KV-SBS process. Spinning solution is continuously carried out from the vessel by sticking on the rolling thread and is subsequently ejected into a jet under the shearing of a high-speed gas flow. The solvents evaporate rapidly, and dry nanofibers are harvested. The thread is refurbished after continuous blow spinning and rolls back into the vessel to maintain perpetual delivery of the solution. (**B**) Photograph of a running KV-SBS equipment. (**C**) High-speed camera image of a jet of solution. The solution wrapped around the nylon thread was shaped into a Taylor cone and then quickly ejected out. The liquid jet experienced an approximately linear motion and then oscillated downstream.

**Fig. 2.**
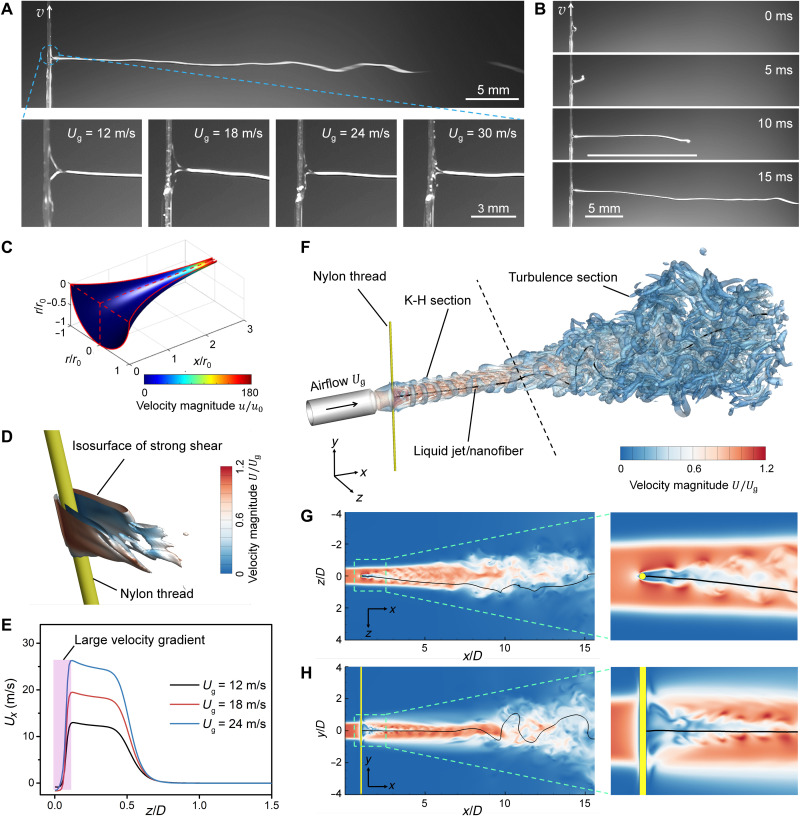
Fiber formation mechanisms. (**A**) The ejected solution captured by a high-speed camera in the KV-SBS process. The cross-sectional area of the Taylor cone shrank as the velocity of airflow increased. (**B**) The liquid jet formation process captured by a high-speed camera. Once delivered to the airflow, the liquid solution was completely shot out as one swinging jet at high speed. (**C**) Predicted cross-sectional radius (*r*) and velocity (*u*) of the liquid jet under gas shearing. *r*_0_ is the initial radius of the cross section, and *u*_0_ is the feeding velocity of the solution (details provided in Supplementary Methods). (**D**) Isosurface of strong shear (norm of fluid strain rate) around the low-speed region on the leeward side of the thread. (**E**) The velocity distribution of *U_x_* along the *z* axis at *x* = 1.1 * *D* at different initial airflow velocities (*U*_g_). (**F** to **H**) CFD simulation results of the airflow velocity magnitude coupled with the swing patterns of the fiber in 3D space (F), *xz* plane at *y* = 0 (G), and *xy* plane at *z* = 0 (H).

The KV-SBS used a newly designed roll-to-roll system to realize needleless solution transport to fabricate nanofibers [Fig. 1, A and B, and movie S1; in our study, polyacrylonitrile (PAN)/*N*,*N′*-dimethylformamide (DMF) solution was used as an example to understand the spinning process and mechanism]. The spinning system was assembled by a roller, a vessel filled with spinning solution, a loop-locked nylon thread, and a gas pipeline set vertically to the thread to provide high-speed air blowing. The spinning solution was continuously carried out via a circulating nylon thread passing through the vessel. The nylon thread was wrapped with spinning solution because of its specific viscosity and a certain degree of wettability with nylon (fig. S2). Driven by the high-speed airflow, the solution was shaped into a Taylor cone and ejected at a high speed, forming nanofibers via fast stretching and swinging, followed by the evaporation of the solvents (Fig. 1C and movies S1 and S2). The size of the Taylor cone decreased with the increase in airflow velocity (Fig. 2A). Randomly distributed nanofibers with the average diameter of as small as 70 nm can be fully harvested at ~30 cm from the spinning point (movie S1 and fig. S3), while the physical behavior of the polymer itself will not obviously change (fig. S4). We meticulously captured the high-speed images at the starting point of spinning and observed that, once delivered to the airflow, the liquid solution was completely shot out as one swinging jet at high speed under gas blowing (Fig. 2B and movie S3). After complete ejection of solution, the nylon thread (fully unwrapped) was refurbished and rolled back to the solution chamber to deliver solution for continuous spinning cycles (fig. S5, A and B). We emphasize that the absence of needles ensured the high stability of the system, enabling long-term operation with minimal maintenance (fig. S5C). In this process, the thread should be kept tight in case that it considerably vibrates, causing unstable jetting and tiny droplets, adversely affecting fiber production. The length of the loop-locked thread should not be too long to reduce the volatilization of the solvents in the transport process and to avoid polymer residues on the thread, especially for solutions with fast evaporation. Sealing of the vessel with a top layer and small holes for nylon to pass through will minimize evaporation of the solvents in the vessel and thereby ensure the stability of the spinning process during long-time operation.

The formation mechanism of nanofibers was investigated by fluidic theory analysis, high-fidelity computational fluid dynamics (CFD) simulations, and bead-spring model for simulating the fiber behavior (the details of the theoretical analysis and models are provided in Supplementary Methods and figs. S6 to S10) (*34*). First, it was pivotal to understand the stability of Taylor cone formed on the leeward side of the thread, which guarantees the formation of continuous and uniform nanofibers. We propose that the high-speed airflow drove the liquid jet in the streamwise direction by gas shearing on the jet surface to form a Taylor cone. Considering that the Taylor cone shape remained unchanged in temporal domain, its cross-sectional area exhibited inverse behavior to that of the magnitude of the liquid jet velocity based on the law of mass conservation of incompressible flow. Therefore, under the driving force of high-speed airflow, the cross-sectional area gradually decreased in the streamwise direction with increasing liquid jet velocity until the cone tip was formed. To theoretically confirm this observation, and to predict the shape function of the cone, we established a one-dimensional (1D) model that took into account the surface tension viscosity of the solution and driving force from the high-speed airflow (further details are provided in Supplementary Methods). The conical shape was predicted by our model, as shown in Fig. 2C.

Second, we propose that the KV effect significantly enhanced the stretching of liquid jet to form ultrathin fibers (detailed discussion in Supplementary Methods). The gas shearing on liquid solution jet holds the key to the formation of uniform fibers with thinner diameters. We emphasize that a large velocity gradient formed on the leeward side of the thread (Fig. 2D). This gradient generated a strong shear stress, enhancing the instability of the liquid-air interface and triggering the liquid jet formation at the initial stage. The velocity gradient was proportional to initial airflow velocity (Fig. 2E), resulting in the size changes of the Taylor cone in Fig. 2A.

Third, we highlighted that the swing amplitude of the liquid jet was strengthened by the KV effect, promoting the solvent evaporation and thus resulting in high-quality nanofibers (Figs. 1C and 2A and movie S2). The 3D vortices of the flow field, visualized by the isosurface of *Q* criterion (*35*), exhibited two distinctive sections after the airflow jet passed the nylon thread (Fig. 2F and movie S4). The first section was barely connected to the thread and was occupied with regularly arranged vortex rings [Kelvin-Helmholtz (K-H) vortices] induced by the K-H instability (*36*). However, unlike the free jet flow without any obstacles, the K-H vortices in this flow configuration were divided into two staggered parts because of the presence of the thread. In this section, denoted as the K-H section, the strong shear stress caused by the high-speed airflow stretched the liquid jet along the streamwise direction and attenuated the swinging amplitude of the jet. Meanwhile, the airflow oscillations induced by the shedding of KVs facilitated the solution evaporation. The second section in the downstream contained massive and unsteady vortices that developed from the transition of the airflow jet upstream. This section was denoted as the turbulence section. We demonstrated that the low-speed regions on the leeward side of the thread with obvious airflow oscillations (Fig. 2, G and H) were induced by the KV street. Because of the unsteady disturbances of the KVs, the interaction between the K-H vortices and KV street promoted the transition of the downstream airflow from laminar into turbulent flow (fig. S11). Unlike the weak swings in K-H section, the liquid jet began to fiercely swing in the turbulence section (Fig. 2, F and G). As a result, solvent evaporation was enhanced, and high-quality nanofibers were obtained.

As a brief summary of the nanofiber fabrication mechanism, the tailor-made flow configuration is a combination of an airflow and a KV street. The high-speed airflow passing across the nylon thread generated strong shear stress and KVs on the leeward side of the thread, beneficial for high-quality nanofiber production. The strong shear stresses stretched the liquid jet in the K-H section, while the KVs disturbed the flow field and triggered an early transition of the airflow from laminar to turbulent flow, promoting the evaporation of the liquid jet. The shape and size of the gas pipeline did not significantly affect the morphologies of the resulting nanofibers, but they could change the distribution of the airflow fields, affecting the motion of the solution jets and the volatilization of the solvents (figs. S12 and S13).

### Universality, tunability, and productivity of KV-SBS

**Fig. 3.**
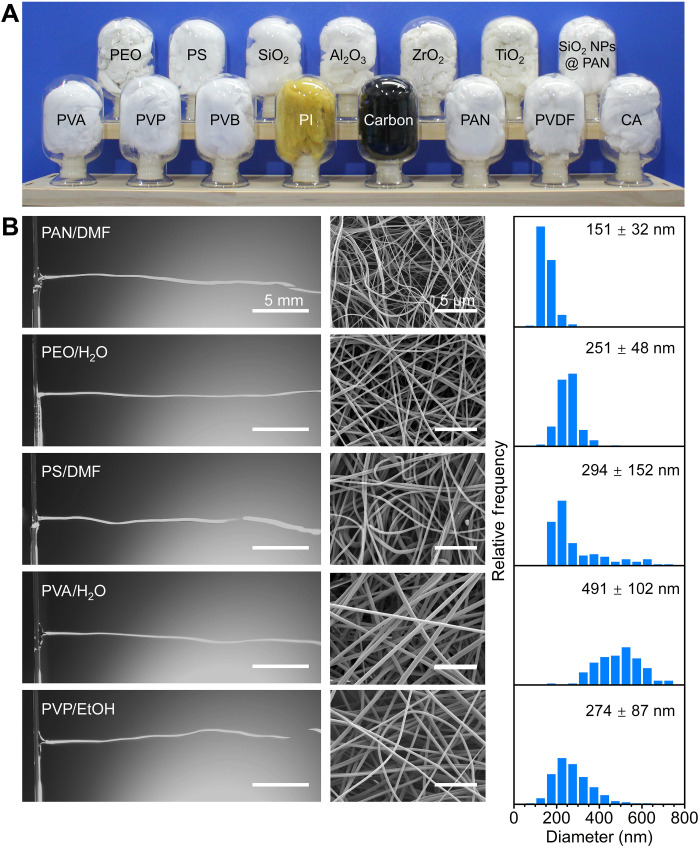
KV-SBS as a generalized strategy to fabricate nanofibers. (**A**) Photographs of a rich variety of nanofiber sponges fabricated by KV-SBS. Detailed fabrication processes are provided in the MATERIALS AND METHODS. SiO_2_ NPs @ PAN, SiO_2_ nanoparticle-loaded PAN nanofibers. (**B**) The ejected liquid jets captured via a high-speed camera in KV-SBS using various polymer solutions (left column) as well as the scanning electron microscope (SEM) images (middle column) and diameter distribution (right column) of corresponding fibers.

To verify the versatility of the KV-SBS strategy, we processed various kinds of nanofibers such as PAN, polyvinyl alcohol (PVA), polyvinyl pyrrolidone (PVP), polyvinyl butyral (PVB), polyimide (PI), polyvinylidene fluoride (PVDF), cellulose acetate (CA), polyethylene oxide (PEO), and polystyrene (PS) as well as carbon, ceramics, and composite nanofibers (Fig. 3A). The spinning processes for these polymer solutions [e.g., PAN/DMF, PEO/H_2_O, PS/DMF, PVA/H_2_O, and PVP/ethanol (EtOH)] were highly stable (the left column in Fig. 3B and movie S5). All the spinning solutions were ejected from the threads by the airflow, forming Taylor cones followed by liquid jet elongation and oscillations as well as accelerated solvent evaporation. The resulting nanofibers had uniform morphologies (center and right columns in Fig. 3B). Carbon nanofibers (fig. S14) and ceramic nanofibers, including SiO_2_, Al_2_O_3_, ZrO_2_, and TiO_2_ nanofibers (fig. S15), were fabricated by postprocessing corresponding precursor fibers, showing potential for applications in high-performance catalyst, sensor, structure reinforcement, and thermal insulation (*37*–*40*). We emphasize the flexibility of the collection of nanofibers in our KV-SBS system for obtaining different fiber-based products such as porous sponges, thin membranes, thick mats, and paper-like materials (fig. S16), ideally suited for various applications such as membrane separation, flexible electronics, and smart textiles (*41*, *42*).

The KV-SBS process is equally efficient in preparing particle-loaded nanofibers without any obstruction. Traditionally, needle-based electrospinning and needle-based SBS methods suffer from needle obstruction if high-concentration solid particles in polymer solution get loaded in the needle hose. KV-SBS faces no such problems because of its needle-free approach from solution delivery to fiber spinning. As a demonstration, we prepared high-content SiO_2_ nanoparticle-loaded PAN nanofibers (SiO_2_ NPs @ PAN) by KV-SBS (Fig. 3A and fig. S17), showing the ability to continuously spin fibers from complex solutions without maintenance. In addition, multicomponent fibers such as PVDF/PAN fiber composites can also be facilely prepared if multiple vessels filled with different spinning solutions are used (fig. S18). Other than nylon threads, we demonstrated many other types of materials, including steel, brass, and cotton threads, as efficient solution delivery systems in our KV-SBS. These threads also fabricated uniform nanofibers with high quality (fig. S19 and movie S6).

The concentration of polymer solution is a critical parameter that can significantly influence the spinning characteristics of the solution and the resulting fiber diameter. We investigated the KV-SBS process using PAN/DMF solutions with different concentrations by a high-speed camera. When the initial concentration was kept low [e.g., 4 weight (wt) %], the solution failed to form a jet under gas shearing, and the formation of tiny individual droplets instead of fibers was observed (fig. S20A). However, at much higher concentrations (e.g., 18 wt %), the polymer chain got entangled, leading to insufficient jet stretching and poor fiber quality (fig. S20D). At moderate concentrations (e.g., 12 wt %), the solution jet experienced a linear motion for a moment before starting to oscillate downstream (fig. S20, B and C).

**Fig. 4.**
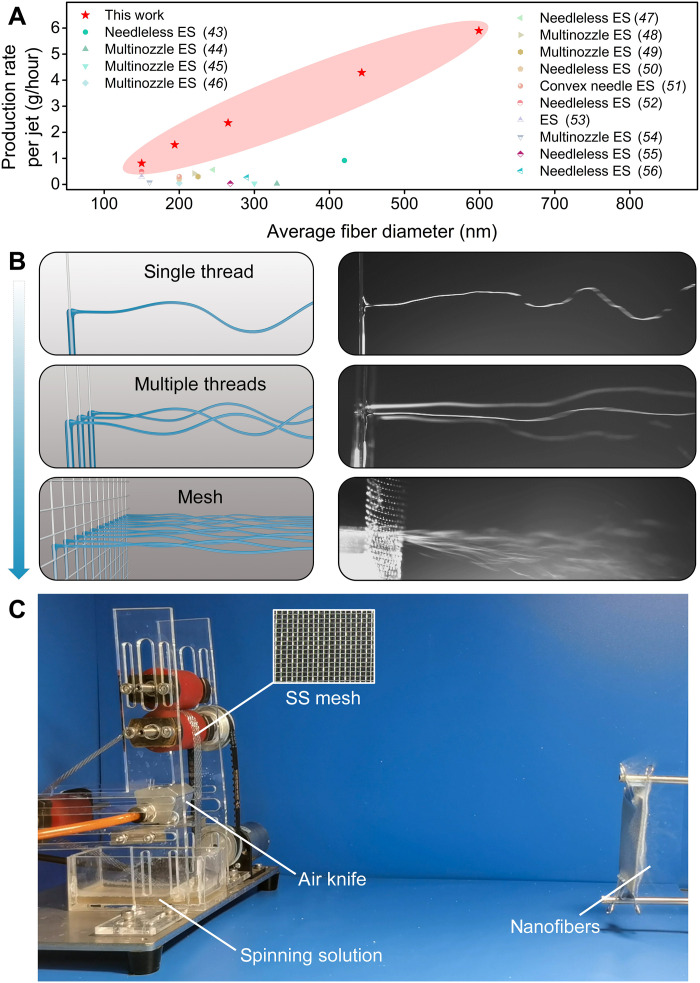
High-throughput production of nanofibers. (**A**) Comparison between KV-SBS and conventional spinning methods in view of production rate per jet and average fiber diameter. ES, electrospinning. (**B**) Schematic diagrams and high-speed camera images of KV-SBS with a single thread, multiple threads, and a mesh. (**C**) Photograph of the KV-SBS system with an SS mesh to deliver solution.

We investigated the effect of the thread speed and the diameter of the gas pipeline (fig. S21, A and B). Then, we fixed the thread speed to 2.5 cm/s to explore the effect of solution concentration on fiber diameter and production rate (fig. S21, C and D). The results showed that higher concentrations resulted in the formation of fibers with larger diameters (figs. S21C and S22). The production rate first increased and then decreased as the solution concentration increased, reaching a peak value of 5.90 ± 0.19 g/hour per thread at a concentration of 16 wt % (fig. S21D). This is consistent with our observations made by the high-speed camera: At high concentrations, it is difficult to elongate the polymer solution because of its high viscosity. The production rates of KV-SBS system were found to be higher than those of many traditional and/or modified electrospinning methods (Fig. 4A and table S1), illustrating the high-throughput potential of this KV-SBS technique for nanofiber production (*43*–*56*).

In addition, the KV-SBS system can easily be reconfigured to host multiple parallel threads (Fig. 4B and movies S7 and S8) and a stainless steel (SS) mesh (Fig. 4B and fig. S23) to improve the fiber production rate and scale. There are several advantages to using a mesh to deliver solution for high-speed spinning: (i) The mesh provides a significantly large density of spinning points, increasing the production rate of nanofibers many fold. (ii) Mesh pore size and density can be easily tuned. We applied commercially available filter screens with different mesh numbers to spin nanofibers (fig. S23). (iii) The KV-SBS system can also accommodate mesh structures with much larger widths. In our experiment, after incorporating SS mesh, a large number of liquid jets shoot out from the large spinning points, continuously forming nanofibers under a sharp gas flow provided by an air knife (Fig. 4, B and C, and movies S9 and S10). Replacing thread with a mesh significantly improved the nanofiber production rate, making the KV-SBS system a powerful platform for industrial-scale production of nanofibers (fig. S24).

**Fig. 5.**
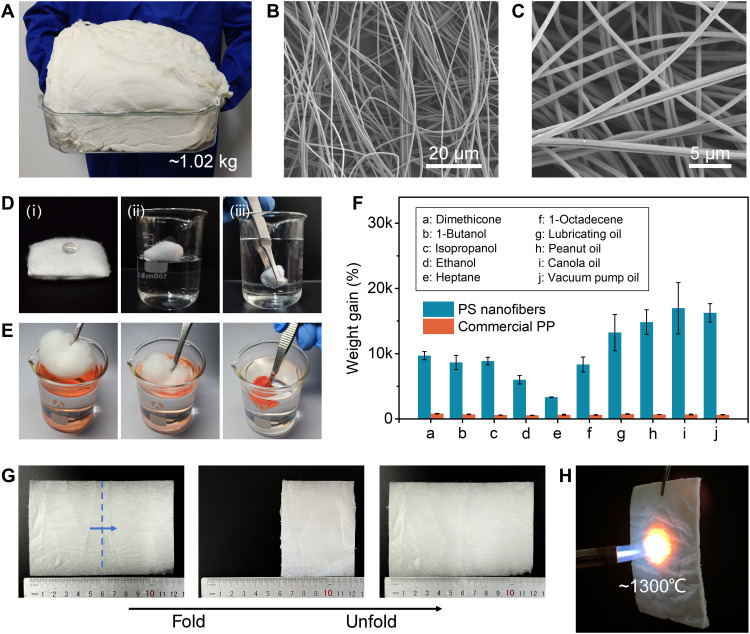
Applications of KV-SBS. (**A**) Photograph of kilogram-scale PS nanofibers fabricated via the KV-SBS process. (**B** and **C**) SEM images of the PS nanofiber sponge. (**D**) Photographic demonstration of hydrophobicity of the PS nanofiber sponge: (i) A PS nanofiber sponge supporting a water droplet. (ii) A PS nanofiber sponge floating on water. (iii) Mirror-like reflection observed when a PS nanofiber sponge was immersed in water. (**E**) Photographs showing the sorption of heptane using a PS nanofiber sponge within 20 s. Heptane was labeled with Sudan red III for clarity. (**F**) Sorption efficiency of the PS nanofiber sponge and a commercial PP oil sorbent for different organic liquids. (**G**) Photographs of a SiO_2_-Al_2_O_3_ fiber mat with foldability. (**H**) A photograph showing fire resistance of the SiO_2_-Al_2_O_3_ fiber mat burned with a butane blowlamp.

The as-fabricated nanofibers achieved high surface areas and can therefore be readily used for separation applications. For example, the 3D porous structure and surface hydrophobicity of PS nanofiber sponge makes it an ideal candidate for the removal of pollutants. Taking advantage of the high-throughput production of nanofibers by the KV-SBS system, we obtained kilogram-scale PS nanofibers with a porous and interlaced 3D structure in a highly efficient manner (Fig. 5, A to C). The PS nanofiber sponge can support a water droplet on its surface and can float on water because it is superhydrophobic and ultralight (Fig. 5D, i and ii). When the sponge was immersed in water, a uniform mirror-like reflection was observed on its surface because of the formation of an interface between the surrounding water and the trapped air in the sponge (Fig. 5Diii). The PS nanofiber sponge exhibited superior absorption capability by completely absorbing the heptane (stained with Sudan red III) within 20 s while floating on a water surface (Fig. 5E). Another nine types of oils and organic solvents were also used to investigate the sorption performance of the PS nanofiber sponge. In general, the PS nanofiber sponges could uptake these organic liquids with a capacity of 33 to 170 times their own weight, outperforming commercial polypropylene (PP) oil-absorbing cotton (Fig. 5F) and many previously reported sorbents (table S2). Moreover, the PS nanofiber sponges exhibited good sustainability and maintained high sorption performance after recycling, although the sorption capacity showed a decline after the first distillation (fig. S25A) and squeezing (fig. S25B).

We also fabricated a flexible SiO_2_-Al_2_O_3_ composite ceramic fiber mat using the KV-SBS technique followed by calcination (Fig. 5G and fig. S26). There was no obvious damage to the appearance of the mat after folding and unfolding (Fig. 5G), demonstrating the flexibility of the mat. The ceramic mat also exhibited good high-temperature resistance and could withstand the flame of a butane blowlamp (Fig. 5H). With these properties, the SiO_2_-Al_2_O_3_ composite ceramic can be useful as insulation and flame retardant. Thus, the KV-SBS strategy demonstrates great potential for the mass production of high-performance ultrathin fibers with a diverse range of applications.

## DISCUSSION

In summary, we developed a versatile and high-throughput KV-SBS method to realize rapid production of various kinds of high-quality nanofibers. The system was built on the basis of the principle of KV street that enables efficient SBS using various types of solution-delivering threads/meshes. We demonstrated that the strong gas shearing assisted in the formation of a Taylor cone and a fast-shooting liquid jet. The interaction between the K-H vortices and the KV street induced by the nylon thread accelerates the transition of the laminar airflow into turbulence downstream, enhancing the swings of the liquid jets and promoting solvent evaporation. This resulted in a production rate as high as 5.90 g/hour per jet. We also demonstrated that this method can be universally applied to fabricate various kinds of fibers and can be easily extended to produce kilogram-scale nanofibers. This needleless KV-SBS system illustrates a new mechanism to blow spin fibers from solution and provides a scalable platform for the industrial production of nanofibers and their broad spectrum of applications.

On the other hand, the theory of KV streets is successfully used to enhance the gas shearing effect and turbulent fluctuations, benefiting the stretching and evaporation of liquid solution jets to obtain high-quality nanofibers. Our strategy materializes the formation of nanofibers via a constructive correlation between the gas flow of the KV street and liquid jet optimization. Moreover, our new insights into the gas-liquid interactions may channelize advanced material processing with numerous potential outcomes in rapid fabrication of water nanodroplets, melt-blown fibers, and solution-driven solid nanopowders.

## MATERIALS AND METHODS

### Fabrication of nanofibers via KV-SBS

#### Polymer nanofibers

Six grams of PAN [weight-average molecular weight (*M*_w_) = 250,000] was dissolved into 44 g of DMF (99.5%) to obtain PAN/DMF solution. Six grams of PVA (PVA-1788) was dissolved into 44 g of deionized water to obtain PVA/H_2_O solution. Six grams of PEO (*M*_W_ = 400,000) was dissolved into 44 g of deionized water to obtain PEO/H_2_O solution. Six grams of PVP [*M*_n_ (number-average molecular weight) = 1,300,000] was dissolved into 44 g of ethanol to obtain PVP/EtOH solution. Five grams of PVB (PVB-50s, Guangdong Yuemei Chemical Co. Ltd.) was dissolved into 45 g of ethanol to obtain PVB/EtOH solution. Four grams of PVDF (PVDF 740, Arkema) was dissolved into 46 g of DMF to obtain PVDF/DMF solution. A total of 7.5 g of PS (*M*_W_ = 280,000) was dissolved into 42.5 g of DMF to obtain PS/DMF solution. Ten grams of PMMA (*M*_W_ = 33,000) was dissolved into 40 g of DMF to obtain PMMA/DMF solution. Six grams of CA (acetyl content, 32.0 wt %; hydroxyl content, 8.7 wt %) and 0.5 g of PEO were dissolved into 44 g of DMF to CA/DMF solution. The polymer solutions were poured into the vessel and spun into corresponding nanofibers, respectively. As for PI nanofibers, a quantity of 0.2 g of PEO was first dissolved into 20 g of DMF. Then, 20 g of polyamide acid (PAA)/DMF solution (20 wt %) was added to the previously prepared PEO/DMF solution. The mixed solution was magnetically stirred at 50°C for 6 hours to obtain a homogeneous solution. Successively, PAA nanofibers were obtained via the KV-SBS process. Subsequently, the PAA nanofibers were treated at 300°C by setting the heating rate to 5°C min^−1^ and by using a holding time of 1 hour. Last, PI nanofibers were obtained.

#### Carbon nanofibers

The PAN nanofibers obtained previously were used as precursors. First, the PAN nanofibers were put in a muffle furnace in an air atmosphere to implement the stabilization process: The nanofibers were heated from room temperature to 230°C by setting the heating rate to 5°C min^−1^. Successively, the heating rate was adjusted to 1°C min^−1^ until the temperature reached 280°C. Afterward, the stabilized PAN nanofibers were carbonized at 1200°C with a heating rate of 5°C min^−1^ and with a holding time of 1 hour in a nitrogen atmosphere. Last, the carbon nanofibers were obtained.

#### SiO_2_ nanofibers

Four grams of PEO was dissolved into 40 g of deionized water by magnetically stirring at 60°C for 1 hour. Then, 15.6 g of tetraethyl orthosilicate (TEOS, 99%) and 0.08 g of H_3_PO_4_ were added into the PEO solution. The solution was magnetically stirred for 6 hours at room temperature. Afterward, the solution was used to prepare precursor nanofiber sponges via the KV-SBS process. The nanofiber sponge was immediately treated at 800°C for 2 hours with a heating rate of 5°C min^−1^ in the air before cooled down in the furnace. Last, the SiO_2_ nanofibers were obtained.

#### Al_2_O_3_ nanofibers

Four grams of PVP was dissolved into a mixture of 20 g of deionized water and 20 g of ethanol. Then, 12 g of aluminum chloride hexahydrate [AlCl_3_·6H_2_O, Analytical Reagent (AR)] was added to the solution. The solution was magnetically stirred for 6 hours at room temperature. Afterward, the homogeneous solution was used to prepare AlCl_3_/PVP nanofiber sponge via the KV-SBS process. The nanofiber sponge was immediately treated at 1100°C for 2 hours with a heating rate of 5°C min^−1^ in the air before cooled down in the furnace. Last, the Al_2_O_3_ nanofibers were obtained.

#### ZrO_2_ nanofibers

Four grams of PVP was dissolved into a mixture of 20 g of deionized water and 20 g of ethanol. Then, 8 g of zirconium oxychloride octahydrate (ZrOCl_2_·8H_2_O, AR) was added to the solution. The solution was magnetically stirred for 6 hours at room temperature. Afterward, the homogeneous solution was used to prepare ZrOCl_2_/PVP nanofiber sponge via the KV-SBS process. The nanofiber sponge was immediately treated at 800°C for 2 hours with a heating rate of 5°C min^−1^ in the air before cooled down in the furnace. Last, the ZrO_2_ nanofibers were obtained.

#### TiO_2_ nanofibers

Four grams of PVP was dissolved into a mixture of 20 g of deionized water and 20 g of ethanol. Then, 16 g of tetrabutyl titanate [Ti(OBu)_4_, 99%] was added to the solution. The solution was magnetically stirred for 6 hours at room temperature. Afterward, the homogeneous solution was used to prepare Ti(OBu)_4_/PVP nanofiber sponge via the KV-SBS process. The nanofiber sponge was immediately treated at 450°C for 2 hours with a heating rate of 5°C min^−1^ in the air before cooled down in the furnace. Last, the TiO_2_ nanofibers were obtained.

#### SiO_2_ NPs @ PAN nanofibers

Six grams of PAN was dissolved into 44 g of DMF to obtain 12 wt % PAN/DMF solution. Then, 3 g of SiO_2_ nanoparticles (30 nm) was added to the solution. The solution was magnetically stirred for 6 hours at room temperature. Afterward, the homogeneous solution was used to prepare SiO_2_ NPs @ PAN nanofibers via the KV-SBS process.

#### PVDF/PAN nanofiber composites

PAN/DMF solution (12 wt %) and PVDF/DMF solution (8 wt %) were poured into two vessels, respectively. Then, they were spun into nanofibers at the same time (see the schematic diagram in fig. S18A), and PVDF/PAN nanofiber composites were obtained.

#### PS nanofiber sponges for oil sorption

Ten grams of PS was dissolved into 40 g of DMF to obtain 20 wt % PS/DMF solution. Afterward, the solution was used to prepare PS nanofibers via the KV-SBS process. A cage-like porous collector was used to collect nanofibers as a sponge.

#### SiO_2_-Al_2_O_3_ composite ceramic fiber mat

A total of 4.5 g of PVA powder was dissolved into 43.2 g of deionized water. Then, 15.6 g of TEOS, 0.08 g of H_3_PO_4_, and 14.4 g of AlCl_3_·6H_2_O were subsequently added to the PVA solution. The solution was magnetically stirred for 6 hours at room temperature. Afterward, the homogeneous solution was used to prepare the precursor fiber mat via the KV-SBS process. The mat was immediately treated at 1000°C for 2 hours with a heating rate of 5°C min^−1^ in the air before cooled down in the furnace. Last, the SiO_2_-Al_2_O_3_ composite ceramic fiber mat was obtained.

### Morphology and structure characterizations

The morphological properties and elementary composition of nanofibers were measured by a field-emission scanning electron microscope (LEO-1530, Zeiss, Germany) equipped with energy-dispersive x-ray spectroscopy. The average fiber diameter was obtained by analyzing 200 U using Image-Pro Plus software (Media Cybernetics, USA). Crystal structure of the carbon and SiO_2_, Al_2_O_3_, ZrO_2_, and TiO_2_ nanofibers were detected by x-ray diffraction (D Max 2500, Rigaku, Japan) test, where the x-ray was Cu-Kα radiation, the scanning speed was 5° min^−1^, and the scanning range was 10° to 80°.

### High-speed camera observation

A high-speed camera (Os7, Integrated Design Tools Inc., USA) equipped with an F-mount lens (atx-i 100 mm F2.8 FF MACRO, Tokina, Japan) was used for the direct observation of the KV-SBS process. A 100-W light-emitting diode lamp (LED-100-T, Visico, China) was used to continuously illuminate the spinning area, which enabled the acquisition of clear images with a short exposure time of the high-speed camera. All the high-speed images were captured at a frame rate of 8000 frames per second (fps), with a picture resolution of 1920 × 416 pixels and an exposure time of 60 μs. The analysis of the videos obtained was carried out using Motion Studio software. All the high-speed videos are played at a frame rate of 24 fps using the images obtained at 8000 fps.

### Sorption capacity measurement

The PS nanofiber sponges were placed in solvents until they were filled with the organic liquid. Then, they were taken out for weight measurement quickly. Here, the sorption capacity was evaluated by weight gain, which is the ratio of the mass of absorbed organics to that of the nanofiber sponge.

## Supplementary Materials

### This PDF file includes:

Supplementary Methods Figs. S1 to S26 Tables S1 and S2 References
