# Supplementary material for: High-throughput production of kilogram-scale nanofibers by Kármán vortex solution blow spinning
Source: Sci Adv. 2022 Mar 16;8(11):eabn3690. doi: 10.1126/sciadv.abn3690 (PMC8926350; doi:10.1126/sciadv.abn3690)
Supplement: Supplementary file 1 — Supplementary Methods Figs. S1 to S26 Tables S1 and S2 References [file sciadv.abn3690_sm.pdf]

Science Advances
AAAS

Supplementary Materials for

High-throughput production of kilogram-scale nanofibers by Kármán vortex solution blow spinning

Ziwei Li, Zhiwen Cui, Lihao Zhao, Naveed Hussain, Yanzhen Zhao, Cheng Yang, Xinyu Jiang, Lei Li, Jianan Song, Baopu Zhang, Zekun Cheng, Hui Wu*

*Corresponding author. Email: huiwu@tsinghua.edu.cn

Published 16 March 2022, *Sci. Adv.* **8**, eabn3690 (2022)
DOI: 10.1126/sciadv.abn3690

The PDF file includes:

Supplementary Methods
Figs. S1 to S26
Tables S1 and S2
Legends for movies S1 to S10
References

Other Supplementary Material for this manuscript includes the following:

Movies S1 to S10

1. Supplementary Methods

1.1 Theoretical analysis of Taylor cone formation

We analysed the formation of the cone in a fixed control volume based on the laws of mass and momentum conservation (fig. S6A). The axial length of the control volume is $\mathrm{d}x$, Q_τ is the external driving force per the unit length caused by the high-speed airflow on the interface and ρ is the density of the solution. $A_1 = \pi r_1^2$ and $A_2 = \pi r_2^2$ are the cross-sectional areas of the inlet and the outlet, respectively, where r_1 and r_2 are the equivalent radius of A_1 and A_2. While U_1 and U_2 are the bulk axial velocities of the inlet and the outlet, respectively. According to the conservation of momentum, we have the following equation:

$$\rho U_2^2 A_2 - \rho U_1^2 A_1 = Q_\tau \mathrm{d}x \qquad \text{Eq. S1}$$

Meanwhile, because of the conservation of mass, the flux of mass Q is expressed as

$$Q = \rho |U_1| A_1 = \rho |U_2| A_2 \qquad \text{Eq. S2}$$

Then we could obtain

$$U_2 - U_1 = \frac{Q_\tau \mathrm{d}x}{Q} \qquad \text{Eq. S3}$$

Here, if Q_τ is always positive, i.e. the external driving force always accelerates the solution, and thus the outlet velocity is larger than the inlet velocity, i.e., $U_2 > U_1$. According to the conservation of mass, the cross-sectional area of the inlet and the outlet satisfies $A_1 > A_2$, and therefore, the equivalent radius gradually reduces as $r_1 > r_2$.

Actually, the shape of the Taylor cone depends on various factors, such as surface tension, the solution viscosity, and the external driving force. To further investigate these effects and predict the cone shape, we proposed a 1D model, as shown in fig. S6B. In this model, the radial velocity variation of the solution jet is ignored, and the driving force from the high-speed airflow on the interface is assumed to be uniformly applied on the cross-section of the solution jet. The 1D Navier-Stokes equation along the streamwise direction is (*57*)

$$u\frac{\mathrm{d}u}{\mathrm{d}x} = -\frac{\gamma}{\rho}\frac{\mathrm{d}}{\mathrm{d}x}\left(\frac{1}{r}\right) + 3\nu\frac{1}{r^2}\frac{\mathrm{d}}{\mathrm{d}x}\left(r^2\frac{\mathrm{d}u}{\mathrm{d}x}\right) + \frac{q_\tau}{\rho} \qquad \text{Eq. S4}$$

Here, u is the bulk velocity of the solution jet, ρ is the solution density, r is the cross-sectional radius, γ is the surface tension coefficient, ν is kinematic viscosity of the solution and q_τ is equivalent driving force per area of cross-section expressed as

$$q_\tau = \frac{0.65\rho_g}{r}\left(U_g - u\right)^2\left[\frac{2\left(U_g - u\right)r}{\nu_g}\right]^{-\beta} \qquad \text{Eq. S5}$$

Here, ρ_g is the density of airflow, U_g is the inlet velocity of the airflow jet and ν_g is the kinematic viscosity of airflow. In order to further simplify the Eq. S4, the viscous term is replaced by $-8\nu\delta u/r^2$ (*58*), where δ is an empirical parameter smaller than one. The simplified equation is expressed as

$$u\frac{\mathrm{d}u}{\mathrm{d}x} = -\alpha\frac{\gamma}{\rho}\frac{\mathrm{d}}{\mathrm{d}x}\left(\frac{1}{r}\right) - \frac{8\delta\nu u}{r^2} + \frac{q_\tau}{\rho} \qquad \text{Eq. S6}$$

Using the radius of the initial cross-section r_0 and the feed velocity of the solution u_0 as characteristic scales, the equation above can be non-dimensionalized as

$$\tilde{u}\frac{\mathrm{d}\tilde{u}}{\mathrm{d}\tilde{x}} = \widetilde{q_\tau} + \frac{2}{We}\frac{1}{\tilde{r}^2}\frac{\mathrm{d}\tilde{r}}{\mathrm{d}\tilde{x}} - \frac{16\delta}{Re}\frac{\tilde{u}}{\tilde{r}^2} \qquad \text{Eq. S7}$$

where the terms with a tilde represent the dimensionless variables. The empirical $\widetilde{q_\tau}$ is

$$\widetilde{q_\tau} = 0.65D_R\tilde{r}^{-1-\beta}\left(\widetilde{U_g} - \tilde{u}\right)^{2-\beta}Re_s^{-\beta} \qquad \text{Eq. S8}$$

Here, $We = \frac{2r_0\rho u_0^2}{\gamma}$, $Re = \frac{2r_0u_0}{\nu}$, $Re_s = \frac{2r_0u_0}{\nu_g}$ and $D_R = \frac{\rho_g}{\rho}$. Due to the dimensionless equation of continuity, $\tilde{r}$ and $\tilde{u}$ satisfy the following constraint:

$$\tilde{r}^2\tilde{u} = 1 \qquad \text{Eq. S9}$$

Differentiating Eq. S9 in the streamwise direction $\tilde{x}$, we obtain

$$-\frac{2}{\tilde{r}^2}\frac{\mathrm{d}\tilde{r}}{\mathrm{d}\tilde{x}} = \frac{1}{\sqrt{\tilde{u}}}\frac{\mathrm{d}\tilde{u}}{\mathrm{d}\tilde{x}} \qquad \text{Eq. S10}$$

By using Eqs. S9 and S10 in Eq. S7, the ordinary differential equation of the solution velocity $\tilde{u}$ is

$$\tilde{u}\frac{\mathrm{d}\tilde{u}}{\mathrm{d}\tilde{x}} = 0.65D_R\tilde{u}^{\frac{1+\beta}{2}}\left(\widetilde{U_g} - \tilde{u}\right)^{2-\beta}Re_s^{-\beta} - \frac{1}{We}\frac{1}{\sqrt{\tilde{u}}}\frac{\mathrm{d}\tilde{u}}{\mathrm{d}\tilde{x}} - \frac{16\delta}{Re}\tilde{u}^2 \qquad \text{Eq. S11}$$

In the same manner, the ordinary differential equation of the shape function of radius $\tilde{r}$ is

$$-\frac{2}{\tilde{r}^5}\frac{\mathrm{d}\tilde{r}}{\mathrm{d}\tilde{x}} = 0.65 D_R \tilde{r}^{-1-\beta}\left(\widetilde{U_g} - \tilde{r}^{-2}\right)^{2-\beta} Re_s^{-\beta} + \frac{2}{We}\frac{1}{\tilde{r}^2}\frac{\mathrm{d}\tilde{r}}{\mathrm{d}\tilde{x}} - \frac{16\delta}{Re}\frac{1}{\tilde{r}^4} \qquad \text{Eq. S12}$$

Note that the solutions of Eqs. S11 and S12 are constrained by Eq. S9. The initial condition of Eqs. S11 and S12 are $\tilde{u}(0) = 1$ and $\tilde{r}(0) = 1$, respectively.

In this study, the coefficient of surface tension γ is 0.07275 N/m for air-water interface. The initial radius of the cross-section $r_0 = 0.001$ m, the feeding velocity of the solution $u_0 = 0.001$ m/s, Weber number $We = 2.75 \times 10^{-5}$, density ratio $D_R = 0.001$, inlet velocity of airflow jet $U_g = 10$ m/s, Reynolds number $Re = 2$ and the corresponding $Re_s = 0.11$. Two empirical parameters β and δ are selected as 0 and 0.5, respectively. To compare the effect of different factors, we calculated the case with $We = \infty$ to eliminate the effect of surface tension while with $Re = \infty$ to eliminate the viscous effect. Fig. S7 shows that the shape function of the cases with $We = \infty$ decreases sharply, while the cases with a finite Weber number decay smoothly. This indicates that surface tension plays a significant role in the cone shape. In addition, according to fig. S7, the viscosity of the solution mainly influences the final radius but does not affect concrete shape of the conical surface. Fig. S8 exhibits the curvature of the shape function $r(x)$ and implies the importance of surface tension on the curvatures of the shape function of the cone.

However, the proposed 1D model qualitatively illustrates the formation of cones. According to Fig. 2B and Movie S3, the droplet-gas interface has undergone a complex process of instability, which eventually leads to the generation of a jet and the formation of a cone. The role that interface instability plays in remains for further exploration. Moreover, the exact expression of the driving force caused by the high-speed airflow is unknown, and the Non-Newtonian effect, i.e. viscoelasticity of the solution, which keeps the solution jet continuous during fiber formation (*59*), might be considered in future.

1.2 Computational fluid dynamics (CFD) simulations

Computation parameters and methods

In order to study the evolution of the airflow field during the spinning process and the airflow effect on the dynamics of the solution jet/nanofiber, we carried out three-dimensional direct numerical simulations (DNSs) by an in-house CFD code with the immersed boundary method (IBM) (*60, 61*). Fig. S9 shows a schematic of the model and computational domain, where *D* is the diameter of the airflow jet nozzle, *d* is the diameter of the circular cylinder (nylon thread), *s* is the distance between the cylinder and the jet nozzle, *L* is the streamwise length of the domain, and *W* and *H* are the width and the height of the computational domain, respectively. We simulated the interaction between the nanofiber and fluid flow after the jet airflow was fully developed. Here, the diameter of the solution jet/nanofiber is approximately 200~500 nm, which is so thin that its feedbacks on the carrier airflow are neglected.

In this study, the Mach number, i.e. the ratio of the speed of airflow to the speed of sound, is much smaller than 0.3 and thus the airflow is considered as incompressible and isothermal. The corresponding Naiver-Stokes and the continuity equations are expressed as,

$$\frac{\partial \boldsymbol{u}}{\partial t} + (\boldsymbol{u} \cdot \boldsymbol{\nabla})\boldsymbol{u} = -\frac{1}{\rho_f}\boldsymbol{\nabla} p + \nu \nabla^2 \boldsymbol{u} + \boldsymbol{f} \qquad \text{Eq. S13}$$

$$\boldsymbol{\nabla} \cdot \boldsymbol{u} = 0 \qquad \text{Eq. S14}$$

Here, $\boldsymbol{u}$ is the velocity vector of airflow, ρ_f is the air density, p is the pressure, ν is the kinematic viscosity of air, and $\boldsymbol{f}$ represents the immersed boundary forces to fulfil the no-slip boundary condition on the cylinder surface.

To implement IBM, the cylinder surface is discretized by uniformly distributed grids. The points of grids are denoted as $\boldsymbol{X}$, which are located on the fixed cylinder boundary. The forces acting on discretized cylinder surface elements are

$$\boldsymbol{F}_L = -\Theta\left[\int_0^t (\boldsymbol{U}_{ib} - \boldsymbol{U})\mathrm{d}t + \Delta t(\boldsymbol{U}_{ib} - \boldsymbol{U})\right] \qquad \text{Eq. S15}$$

where Θ is a large constant, $\Delta \mathrm{t}$ is the computational time step of flow, $\boldsymbol{U}$ represents the velocity of fixed points $\boldsymbol{X}$ on the cylinder surface and always equals zero during computation, and $\boldsymbol{U}_{\mathrm{ib}}$ is the

fluid velocity of points $\boldsymbol{X}$ interpolated from the fluid velocity located on the surrounding Eulerian grid points $\boldsymbol{x}$ through a delta function, i.e.,

$$\boldsymbol{U}_{ib} = \sum \boldsymbol{u} \delta_h (\boldsymbol{X} - \boldsymbol{x}) \Delta V \qquad \text{Eq. S16}$$

Here $\Delta \mathrm{V} = h^3$, where h is the size of grid cell. Note that the Cartesian grids around the cylinder surface are uniform with the size h in all three directions. In addition, the delta function is expressed as

$$\delta_h(x) = \frac{1}{h^3} \phi\left(\frac{x}{h}\right) \phi\left(\frac{y}{h}\right) \phi\left(\frac{z}{h}\right) \qquad \text{Eq. S17}$$

where

$$\phi(r) = \begin{cases} \frac{1}{6}\left(5 - 3|r| - \sqrt{2 + 6|r| - 3r^2}\right), & 0.5 < |r| \leq 1.5, \\ \frac{1}{3}\left(1 + \sqrt{-3r^2 + 1}\right), & |r| \leq 0.5, \\ 0, & \text{otherwise} \end{cases} \qquad \text{Eq. S18}$$

denotes the three-point smoothed delta function (*62*). In turn, the forces $\boldsymbol{F}_{\mathrm{L}}$ on the cylinder surface are spread onto the Eulerian grids to compute forces $\boldsymbol{f}$, i.e.

$$\boldsymbol{f} = \sum \boldsymbol{F}_L \delta_h (\boldsymbol{x} - \boldsymbol{X}) \Delta V \qquad \text{Eq. S19}$$

Concerning the flow solver, we employed an implicit velocity decoupling procedure on staggered Cartesian grid system for the incompressible Navier-Stokes equations (*63*). The computational domain of the whole fluid field is $20D \times 8D \times 8D$ for L, W and H, respectively. The characteristic scale of length is D = 4 mm, while the diameter of the cylinder d is 0.1D, and the distance between the inlet and the centre of cylinder s is equal to D. We use 96 uniform grids per length D. Therefore, the total number of grids is approximately 1.13 billion. We applied periodic boundary conditions for velocity and pressure in both y and z directions. At the inlet, the velocity of the jet nozzle is given by a top-hat function (*64*), i.e.

$$\boldsymbol{u} = \left\{\frac{U_g}{2} - \frac{U_g}{2} \tanh\left[\frac{1}{8}\frac{D}{\delta}\left(\frac{2\xi}{D} - \frac{D}{2\xi}\right)\right]\right\} \boldsymbol{e}_x \qquad \text{Eq. S20}$$

Here, δ_m is the momentum thickness, the ratio $D/\delta_m = 40$, U_g is the axial bulk velocity of jet flow, $\xi = \sqrt{(y-y_0)^2 + (z-z_0)^2}$ is the distance from the jet origin and $\boldsymbol{e}_x$ is the unit vector corresponding to the streamwise direction. We imposed the convective boundary condition at the outlet. In addition, the kinematic viscosity of airflow is $1.48\times 10^{-5} \mathrm{m}^2/\mathrm{s}$, and the velocity of the jet near the nozzle is 7.4 m/s. Therefore, the corresponding Reynolds number of the airflow jet is $Re = U_g D/\nu_g = 2000$.

To investigate the effect of the Kármán vortex street on the airflow jet transition, we performed two direct numerical simulations with and without the nylon thread. The inlet velocities of these two simulations are both imposed by a top-hat function, i.e., Eq. S20, with 5% Gaussian white noises. In fig. S11, the relatively stable high-speed section in panel C is obviously longer than that in panels A and B, which clearly reflects that the presence of the thread induces the Kármán vortex street that promotes the transition of the airflow jet from laminar to turbulent.

<u>Bead-spring model for nanofiber</u>

To simulate the motions of the solution jet/nanofiber connected to the thread (fig. S9), we used a bead-spring model of the solution jet/nanofiber (fig. S10, A and B) (*34*). $\mathbf{q}_i$ is the vector from the origin to the centre of each bead, and $\boldsymbol{t}_i$ is the tangent vector as $\boldsymbol{q}_i - \boldsymbol{q}_{i-1}$. Therefore, the principal stretching force of each bead $\boldsymbol{F}_i^s$ is

$$\boldsymbol{F}_i^s = \boldsymbol{T}_i^1 + \boldsymbol{T}_i^2 = -k\left(1 - \frac{l_0}{|\boldsymbol{t}_i|}\right)\boldsymbol{t}_i + k\left(1 - \frac{l_0}{|\boldsymbol{t}_{i+1}|}\right)\boldsymbol{t}_{i+1} \qquad \text{Eq. S21}$$

where *k* is Hooke's coefficient and l_0 is the equilibrium distance of the beads.
For the beads, their governing equation is

$$m_i \dot{\boldsymbol{v}}_i = \boldsymbol{F}_i^s + \boldsymbol{F}_i^h, \text{where } \boldsymbol{F}_\mathrm{i}^h = 6\pi\mu a(\boldsymbol{u}_i - \boldsymbol{v}_i) \qquad \text{Eq. S22}$$

$$\dot{\boldsymbol{q}}_\mathrm{i} = \boldsymbol{v}_i \qquad \text{Eq. S23}$$

Here, m_i is the mass of the *i*-th bead, *a* is the equivalent radius of the bead, $\boldsymbol{v}_\mathrm{i}$ is the velocity of the *i*-th bead, and $\boldsymbol{u}_i$ is the fluid velocity at the *i*-th bead, which is interpolated from the neighbouring grids by a second-order Lagrangian interpolation. $\boldsymbol{F}_i^h$ is the hydrodynamic drag force on the *i*-th bead.

Meanwhile, the jet is continuously ejected from the droplet and the fiber formed from the jet is transported downstream with the flow. To simulate this process, we set the length of the fiber modelled by the bead-spring model as 5 times longer than L to ensure the length of the valid section of fiber during the computation as shown in fig. S10C. The start point of the valid section is at $(x = s + 0.5d, y = 0, z = 0)$, while the end point is at $x = L$. In the outlet section, the velocities of fluid at beads are assumed to be equal to the values at the end of the valid section to avoid the accumulation of fiber around the outlet. In addition, we used 32 beads per D to discretize the fiber. Meanwhile, the Young's modulus of fiber is E = 42.8 GPa, and the diameter of fiber $d_f = 500\ \mathrm{nm}$. Therefore, the equivalent Hook's coefficient k is

$$k = \frac{\pi}{4} E d_f^2 \frac{N_0}{D} \approx 67.2 \frac{\mathrm{N}}{\mathrm{m}} \qquad \text{Eq. S24}$$

where N_0 is the number of beads per D, i.e. $\mathrm{N}_0 = 32$. The equivalent radius a of bead is

$$a = \left(\frac{3}{16} d_f^2 \frac{D}{N_0}\right)^{\frac{1}{3}} \approx 1.80\mu\mathrm{m} \qquad \text{Eq. S25}$$

2. Supporting Figures

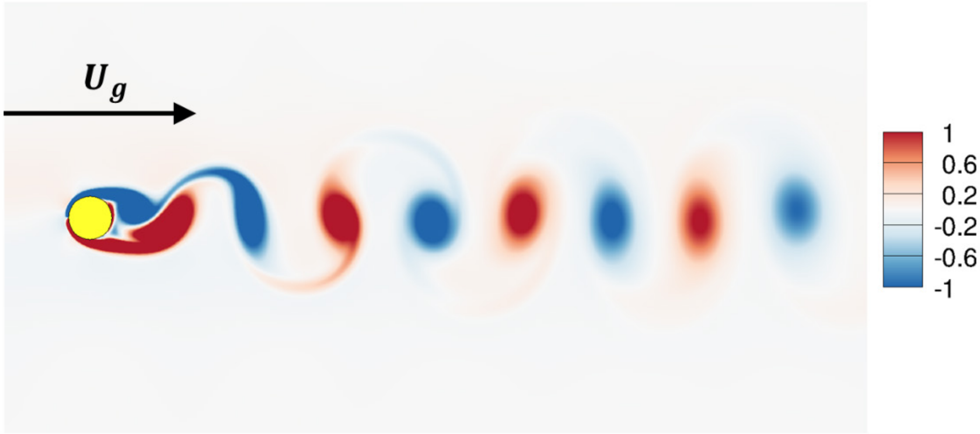

Fig. S1. A Schematic diagram of Kármán vortex street. It is generated by uniform flow over a cylinder with $Re_d = U_g d/\nu = 200$, where U_g is the inlet velocity, d is the diameter of cylinder and ν is the kinematic viscosity of fluid. The color represents the vorticity $\omega_z d/U_g$. When a uniform flow passes through a bluff body beyond a critical Reynolds number Re_d, a pair of vortices unceasingly formed on the leeward side of the bluff obstacle are shedding periodically and transported downstream. The clockwise and counter-clockwise vortices are arranged to lines and formed the so-called Kármán vortex street. For flow passing over a circular cylinder, the vortex street appears when $Re_d = U_g d/\nu$ is over 47.

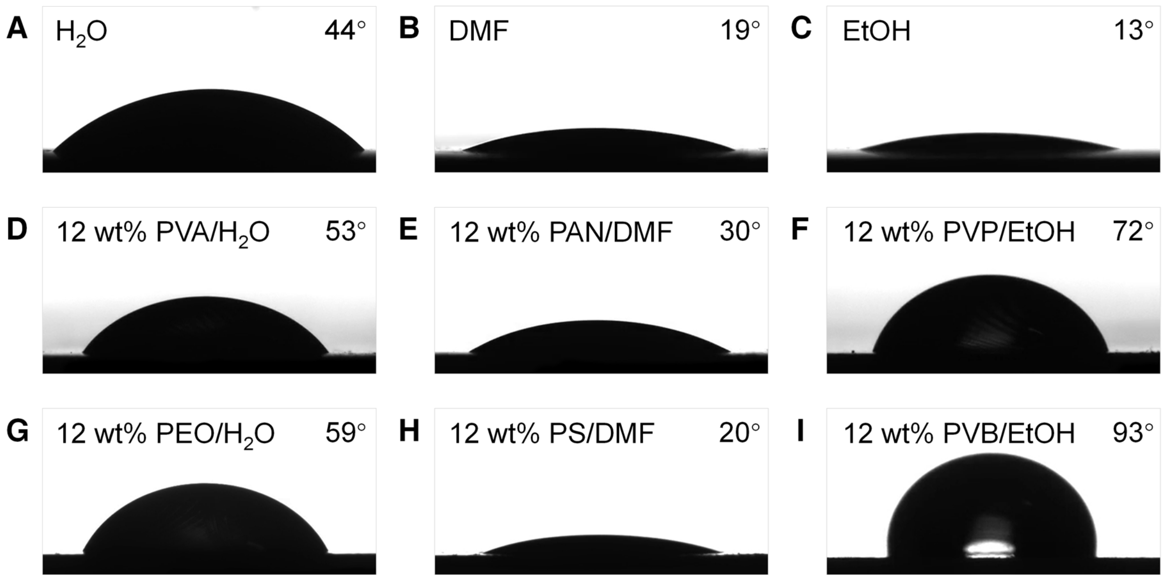

Fig. S2. The wettability of nylon sheet with different solvents/solutions. (**A**) Water. (**B**) DMF. (**C**) Ethanol (EtOH). (**D**) 12 wt% PVA/H_2O. (**E**) 12 wt% PAN/DMF. (**F**) 12 wt% PVP/EtOH. (**G**) 12 wt% PEO/ H_2O. (**H**) 12 wt% PS/DMF. (**I**) 12 wt% PVB/EtOH.

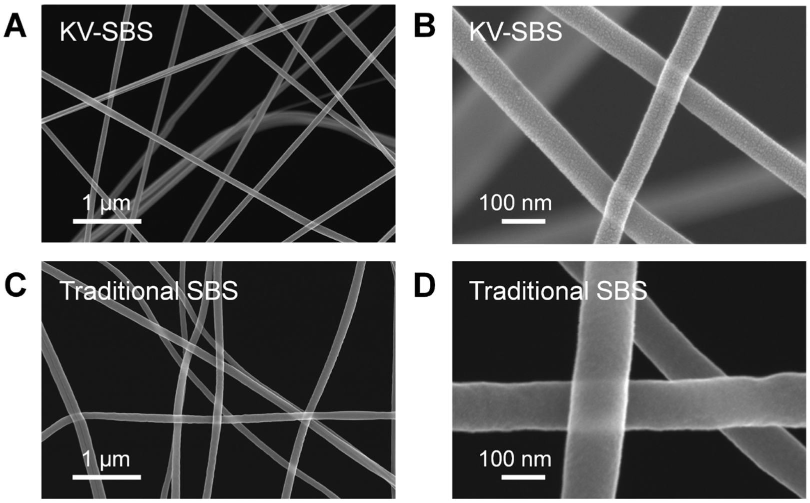

Fig. S3. Comparison between nanofibers fabricated via KV-SBS and traditional SBS. (**A, B**) SEM images of PAN nanofibers fabricated via KV-SBS. The average fiber diameter is as small as ~ 70 nm. (**C, D**) SEM images of PAN nanofibers fabricated via traditional SBS using the same spinning solution. The average fiber diameter is ~ 120 nm. This demonstrates the ability of the KV-SBS technique to fabricate ultra-fine nanofibers.

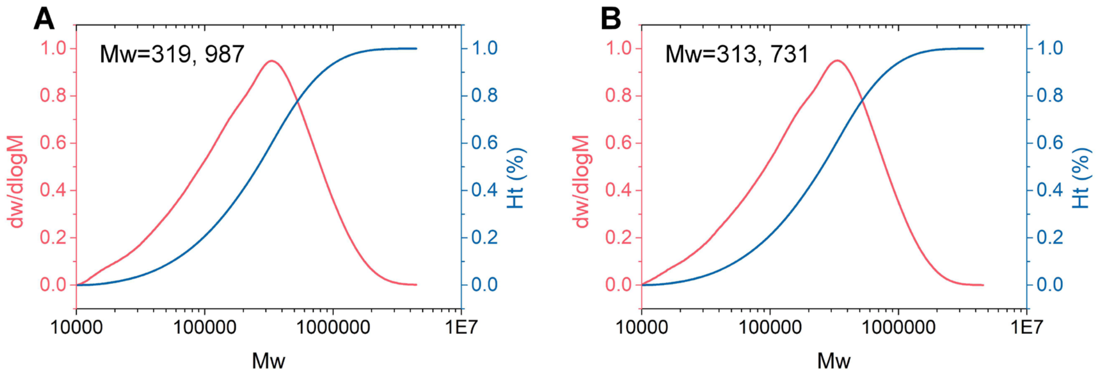

Fig. S4. Molecular weight distribution of PAN pre- and post-spinning measured by gel permeation chromatography (GPC). (**A**) PAN powder (before spinning) (**B**) PAN nanofibers (after spinning). There was no obvious change in the distribution plots of the polymers, which demonstrates the similarity in their physical behavior.

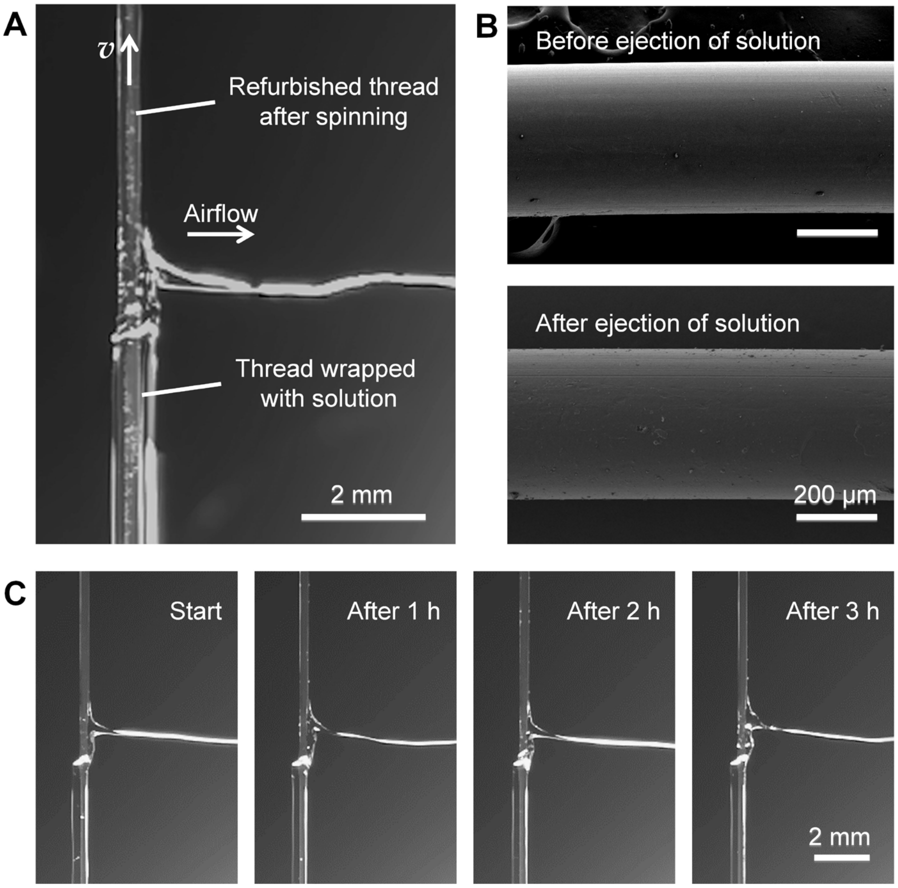

Fig. S5. Characterization of the nylon thread in the KV-SBS process. (**A**) High-speed camera image of a jet. The solution wrapped around the nylon thread was quickly ejected out. After complete ejection of the solution, the nylon thread was refurbished. (**B**) SEM images of the thread before and after ejection of spinning solution. (**C**) High-speed camera images of the KV-SBS process during a long-time operation, demonstrating the stability of the system.

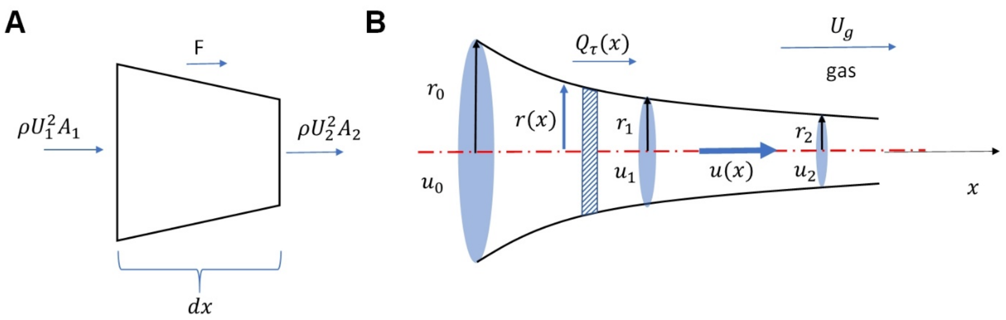

Fig. S6. Theoretical analysis of Taylor cone formation. (**A**) A fixed control volume based on the laws of mass and momentum conservation. (**B**) Schematic of one-dimensional model.

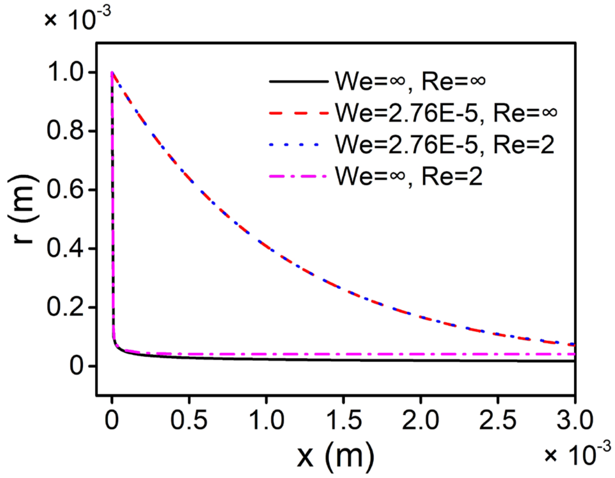

Fig. S7. Shape function $r(x)$ with different settings. Here, $\mathrm{We} = \infty$ means that surface tension is neglected while $\mathrm{Re} = \infty$ represents that viscous effect is neglected.

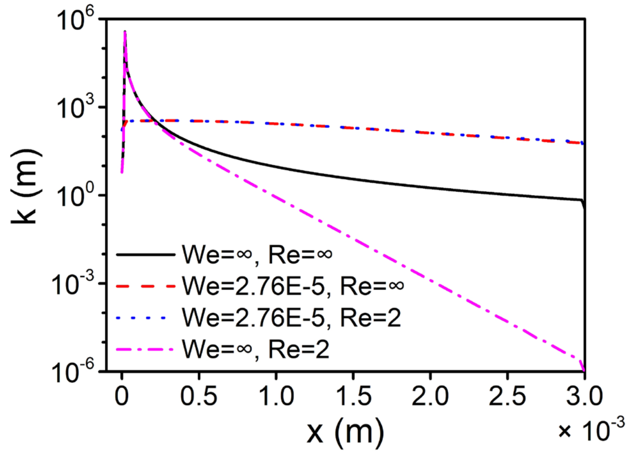

Fig. S8. Curvature of the shape function with different settings. Here, $We = \infty$ means that surface tension is neglected while $Re = \infty$ represents that viscous effect is neglected.

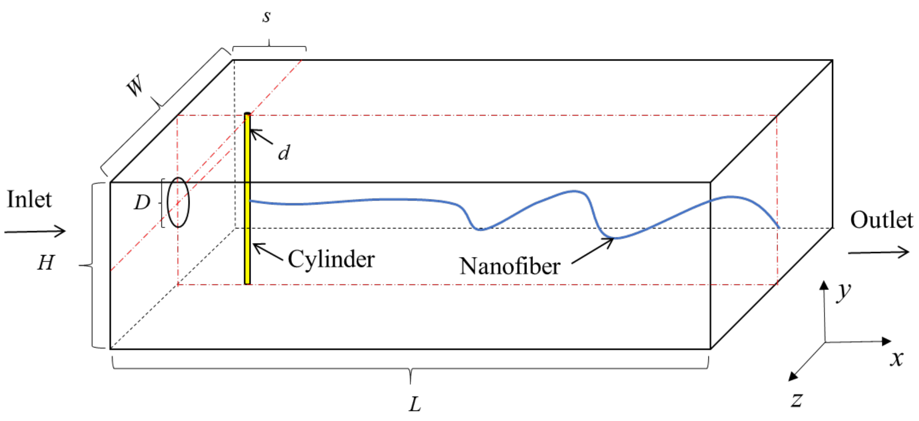

Fig. S9. Sketch of the computational domain.

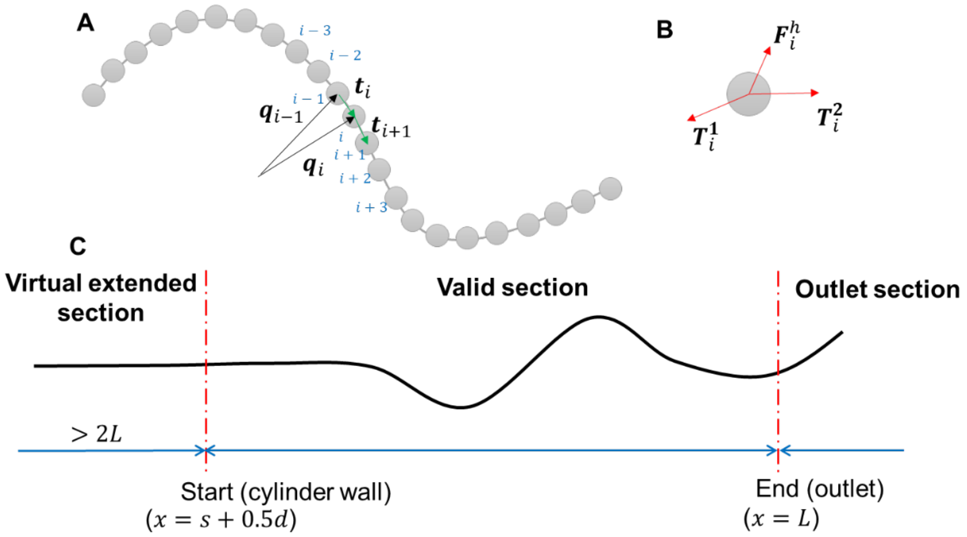

Fig. S10. Schematic of the bead-spring model.

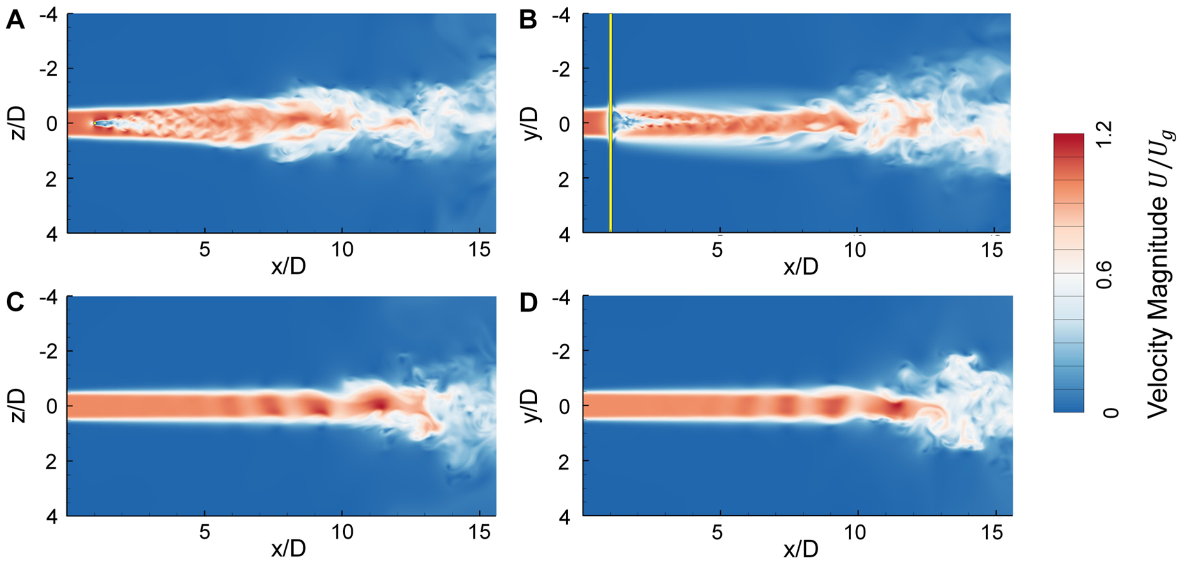

Fig. S11. Investigation into the effect of Kármán vortex street on the airflow jet transition. (**A, B**) Velocity magnitude contours of an airflow jet passing over the thread in x-z and x-y planes. (**C, D**) Velocity magnitude contours of an airflow jet without the thread in x-z and x-y planes.

The inlet velocities of these two simulations are both imposed by a top-hat function, i.e. Eq. S20, with 5% Gaussian white noises. The relatively stable high-speed sections in panel C and D are longer than that in panels A and B, demonstrating that the transition of the downstream airflow jet from laminar into turbulent flow is promoted by the Kármán vortex street.

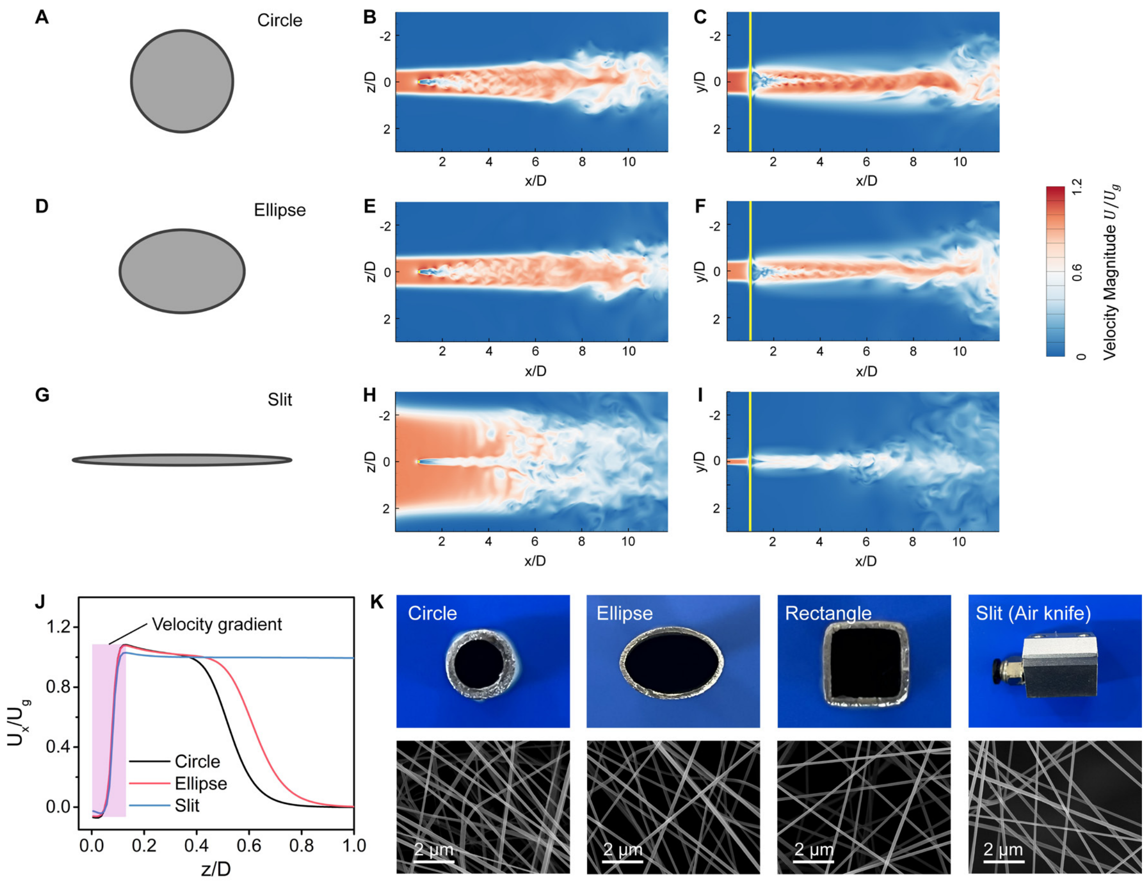

Fig. S12. Investigation into the effect of the shape of gas pipeline. (**A**) Schematic diagram of a circular gas pipeline. Its diameter is D. (**B, C**) Velocity magnitude contours of an airflow jet from the circular pipeline in x-z and x-y planes when passing over the thread. (**D**) Schematic diagram of an elliptic gas pipeline. Its major to minor axis ratio is 1.5. Its area is $\pi D^2/4$. (**E, F**) Velocity magnitude contours of an airflow jet from the elliptic pipeline in x-z and x-y planes when passing over the thread. (**G**) Schematic diagram of a slit gas pipeline. Here, an ellipse with its major to minor axis ratio of 20 was used to approximate the slit. Its area is $\pi D^2/4$. (**H, I**) Velocity magnitude contours of an airflow jet from the slit pipeline in x-z and x-y planes when passing over the thread. (**J**) The velocity profile of U_x along the z axis at x = 1.1 * D under airflows from pipelines with different shapes. The diameter of the cylinder d is 0.1D. The change of pipeline diameter didn't significantly affect the shear stress on the leeward side of the thread. (K) SEM images of PAN nanofibers fabricated via KV-SBS using gas pipelines with different shapes. Each of the resulting nanofibers had uniform morphologies.

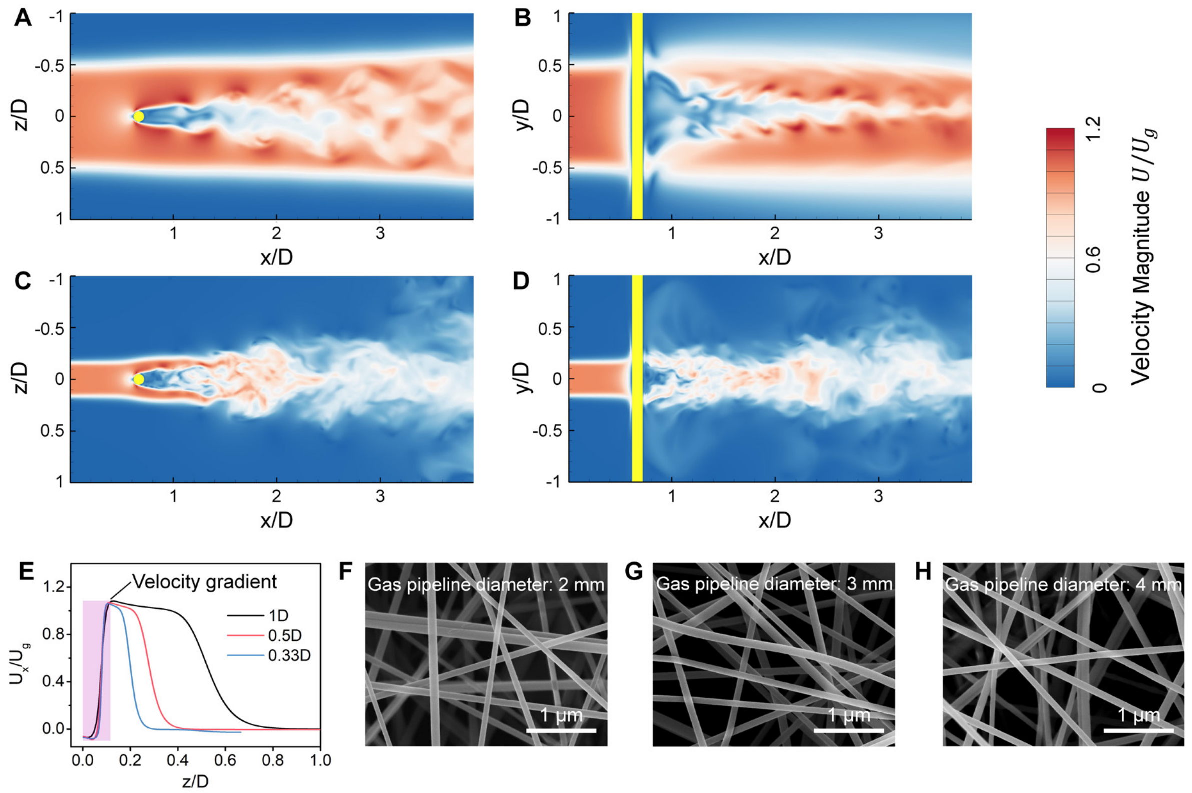

Fig. S13. Investigation into the effect of the diameter of gas pipeline. (**A, B**) Velocity magnitude contours of an airflow jet (the diameter is D) passing over the thread in x-z and x-y planes. D equals to 4 mm. (**C, D**) Velocity magnitude contours of an airflow jet (the diameter is 0.33*D) passing over the thread in x-z and x-y planes. (**E**) The velocity profile of U_x along the z axis at x = 1.1 * D under airflows with different diameters (1*D, 0.5*D, and 0.33*D). The diameter of the cylinder d is 0.1D. The change of pipeline diameter did not significantly affect the shear stress on the leeward side of the thread. (**F–H**) SEM images of PAN nanofibers fabricated via KV-SBS. The inner diameter of the gas pipeline is 2 mm (**F**), 3 mm (**G**), and 4 mm (**H**), respectively. The morphologies of the nanofibers did not significantly change with the pipeline diameter.

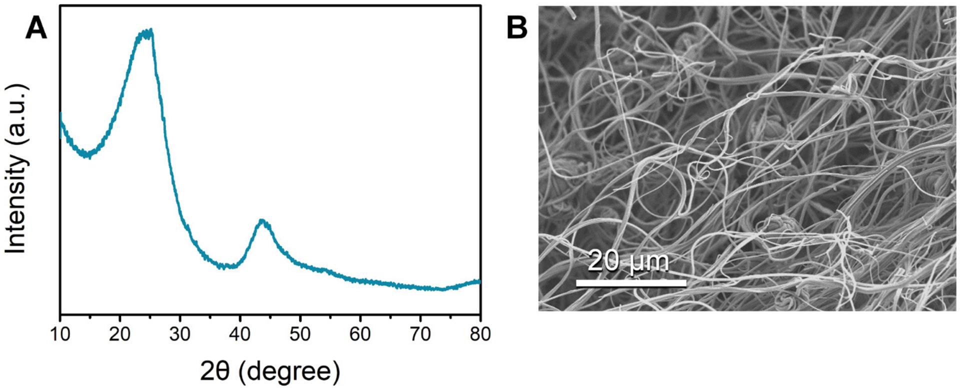

Fig. S14. Characterization of Carbon nanofibers fabricated via the KV-SBS process. (**A**) XRD pattern of the Carbon nanofibers. (**B**) SEM image of the Carbon nanofibers.

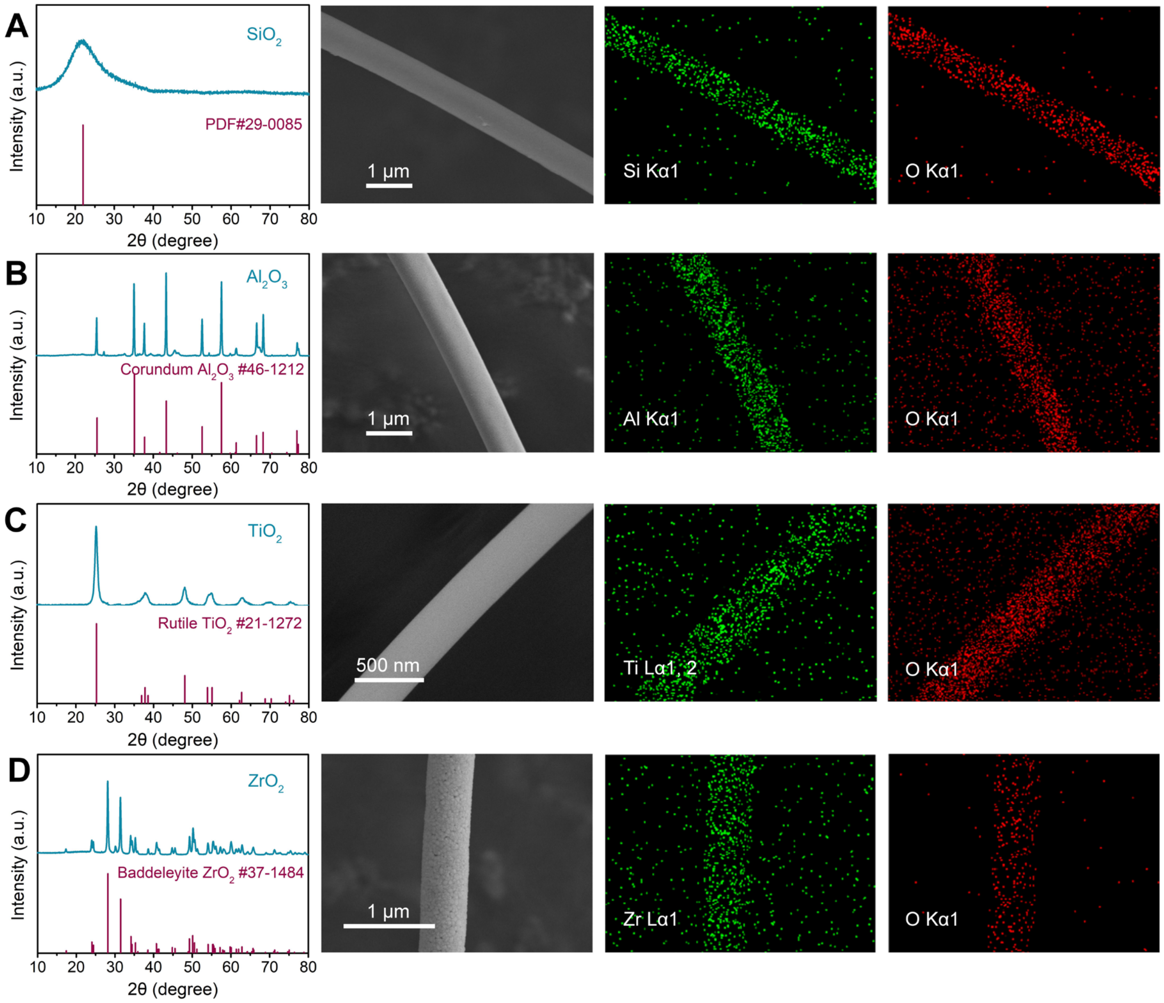

Fig. S15. Characterization of ceramic nanofibers fabricated via the KV-SBS process. (**A-D**) XRD, SEM pattern and corresponding elemental mapping images of the SiO_2 (A), Al_2O_3 (B), TiO_2 (C), and ZrO_2 (D) nanofibers fabricated via the KV-SBS process.

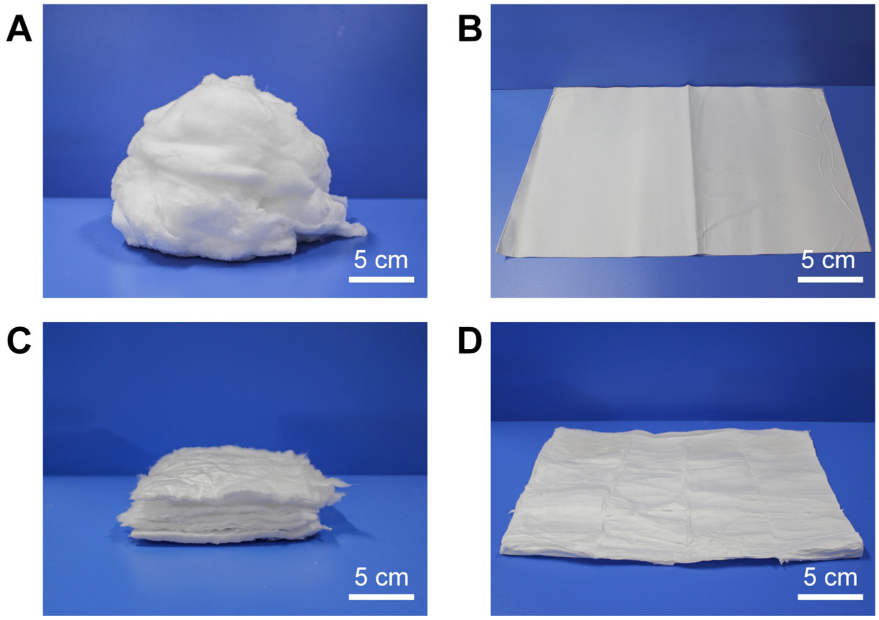

Fig. S16. Photographs of different nanofiber-based products fabricated via the KV-SBS process. (**A**) Porous sponges. (**B**) Thin membranes. (**C**) thick mats. (**D**) paper-like materials.

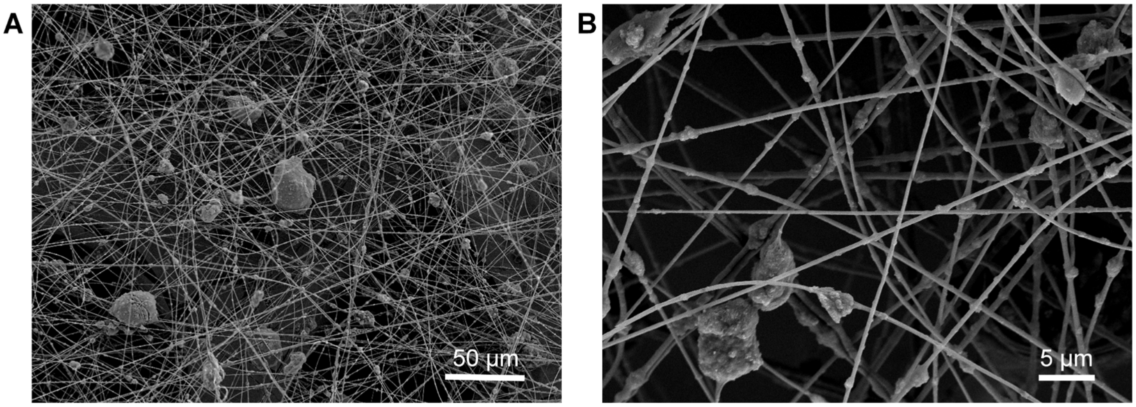

Fig. S17. SEM images of SiO_2 NPs @ PAN nanofibers.

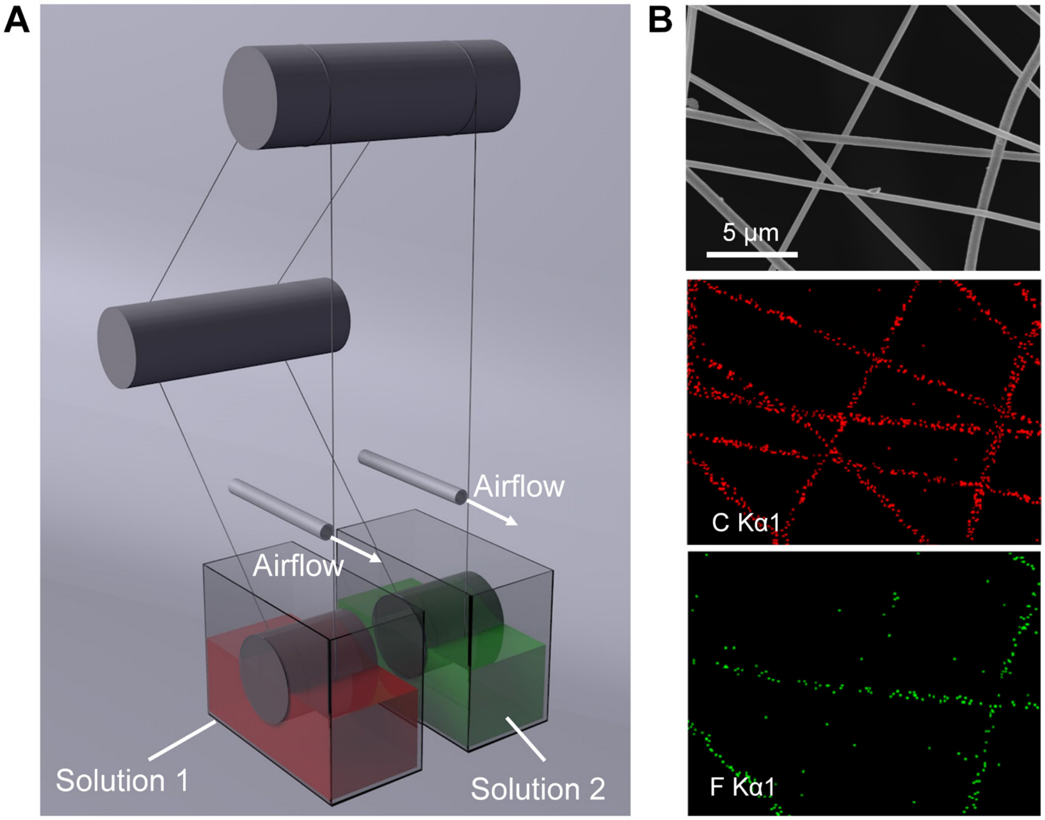

Fig. S18. Fabrication of composite nanofibers via KV-SBS. (**A**) Schematic diagram of KV-SBS for the preparation of bicomponent nanofibers. (**B**) SEM image of PAN/PVDF nanofibers and corresponding elemental mappings of Carbon and Fluorine.

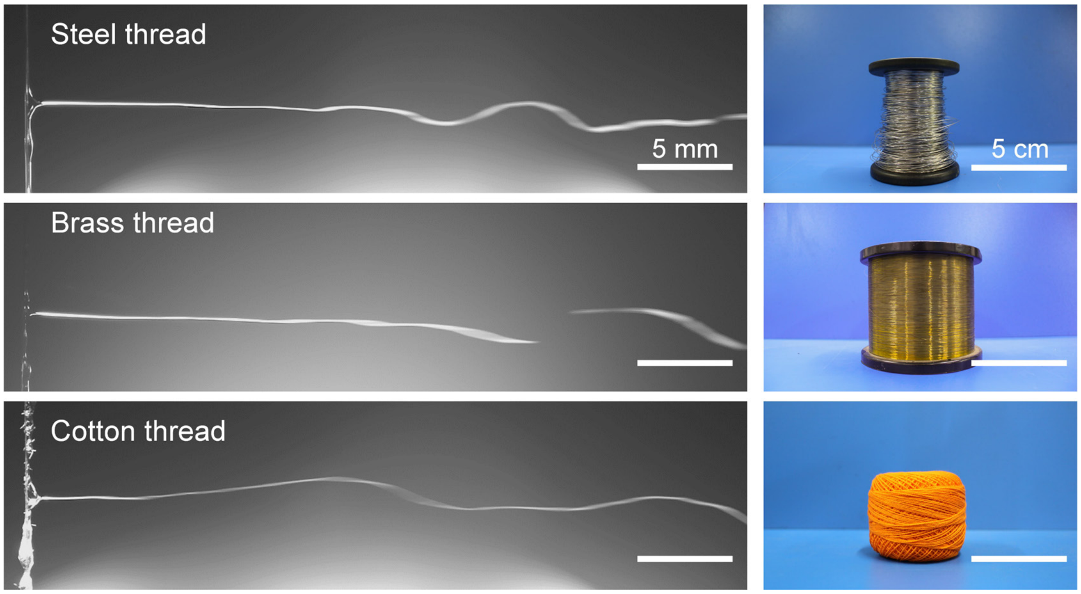

Fig. S19. High-speed camera images of the KV-SBS process with different threads and photographs of the threads.

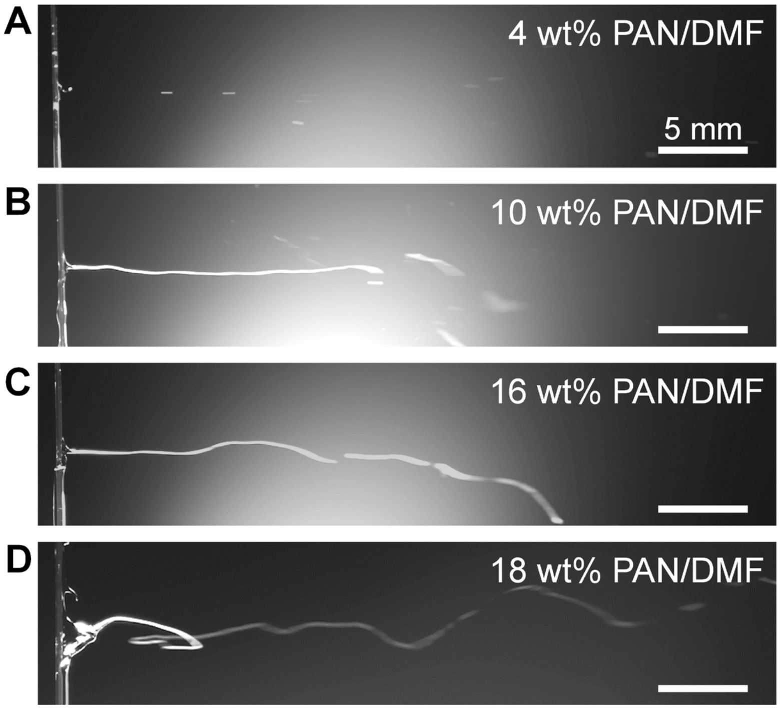

Fig. S20. High-speed camera images of the KV-SBS process for different concentrations of PAN/DMF solutions. (**A**) 4 wt%. (**B**) 10 wt%. (**C**) 16 wt%. (**D**) 18 wt%.

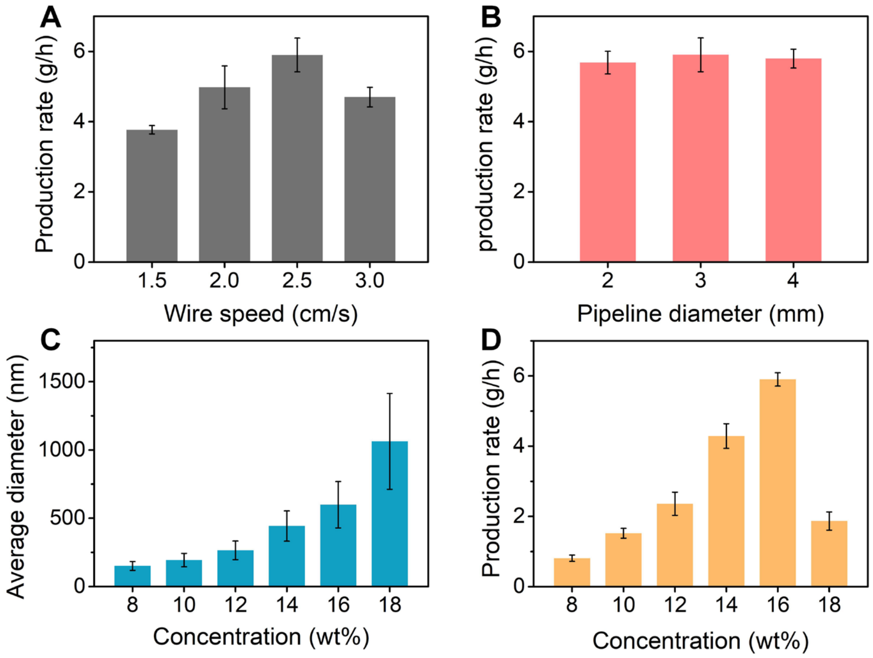

Fig. S21. Production rates of KV-SBS. (**A**) Production rates of PAN nanofibers at different thread speeds. The concentration of PAN / DMF solution is 16 wt%. (**B**) Production rate of PAN nanofibers using gas pipelines with different inner diameters. The concentration of PAN / DMF solution is 16 wt%. The thread speed is 2.5 cm/s. (**C, D**) Average diameter (**C**) and production rate (**D**) of PAN nanofibers using different solution concentrations at a thread speed of 2.5 cm/s. The inner diameter of the gas pipeline is 3 mm.

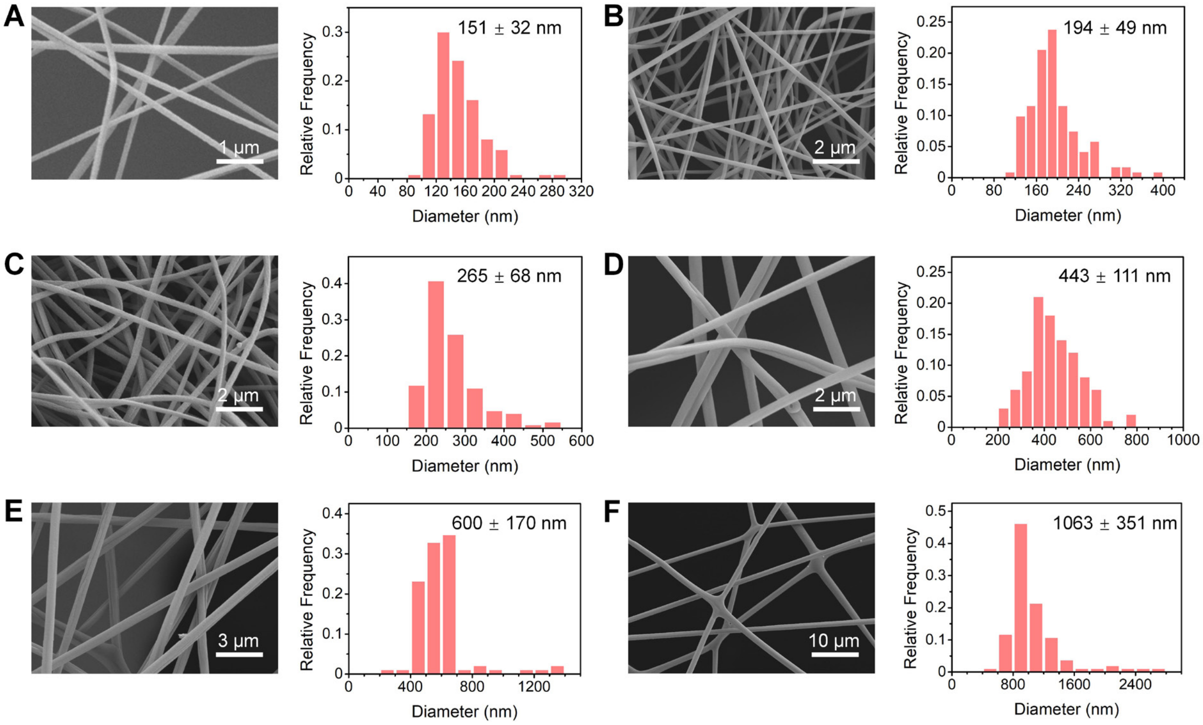

Fig. S22. SEM images and diameter distribution statistics of PAN nanofibers prepared from solutions of different concentrations. (**A**) 8wt%. (**B**) 10wt%. (**C**) 12wt%. (**D**) 14wt%. (**E**) 16wt%. (**F**) 18wt%.

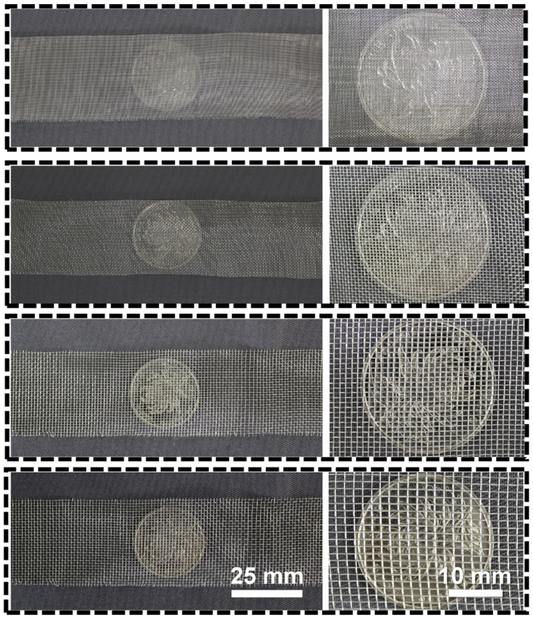

Fig. S23. Meshes with different mesh numbers for the KV-SBS process.

A

Mesh number	Mesh aperture size/mm	Mesh wire diameter/mm
12	1.80	0.30
30	0.67	0.18
50	0.38	0.13
100	0.18	0.08

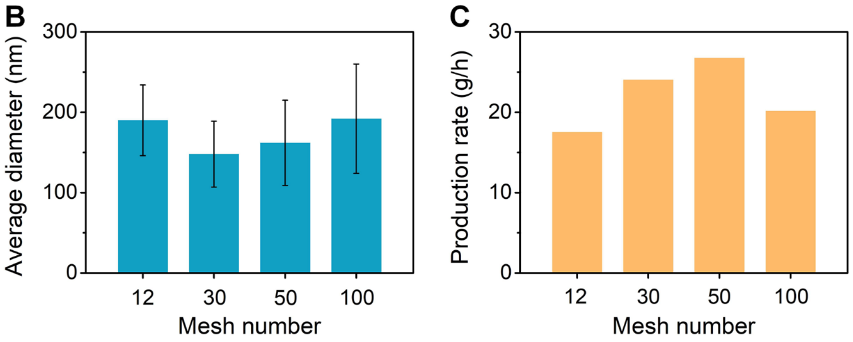

Fig. S24. Investigation into meshes with different mesh numbers for the KV-SBS process. (A) Aperture sizes and wire diameters of different meshes. **(B, C)** Average diameters and production rates of nanofibers fabricated by different meshes. The width of each mesh is 2 cm. 12wt% PAN/DMF solution was used.

Meshes with larger mesh numbers (smaller aperture sizes) can load more spinning solutions and provide larger density of spinning points, promoting the production of nanofibers. On the other hand, smaller aperture size leads to greater air resistance and thereby, inadequate drafting of solution jets. This would result in a decrease in production rate of nanofibers when the aperture size is very small.

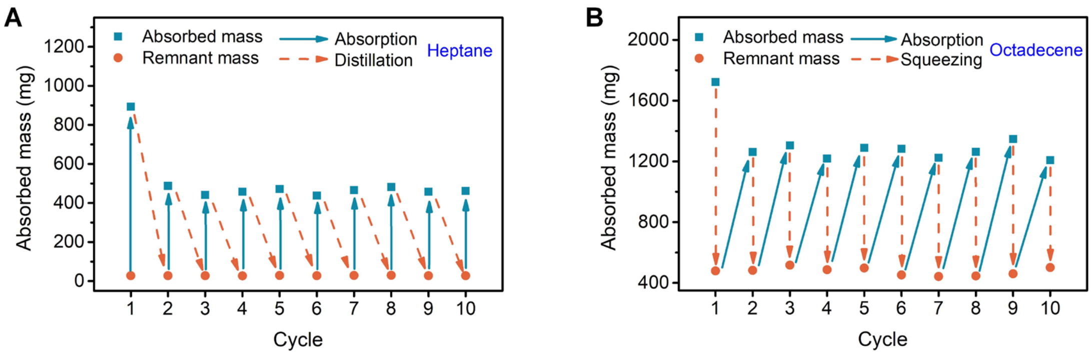

Fig. S25. Sustainability of PS nanofiber sponges for oil sorption. (**A**) Distillation was used to recycle the PS nanofiber sponge for sorption of heptane. The sponge absorbed solvents far exceeding its own weight and maintained high sorption performance after 10 cycles of the sorption-distillation process. (**B**) Squeezing was applied to recycle the PS sponge for sorption of octadecene. The sponge maintained high sorption performance after 10 cycles of the sorption-squeezing process.

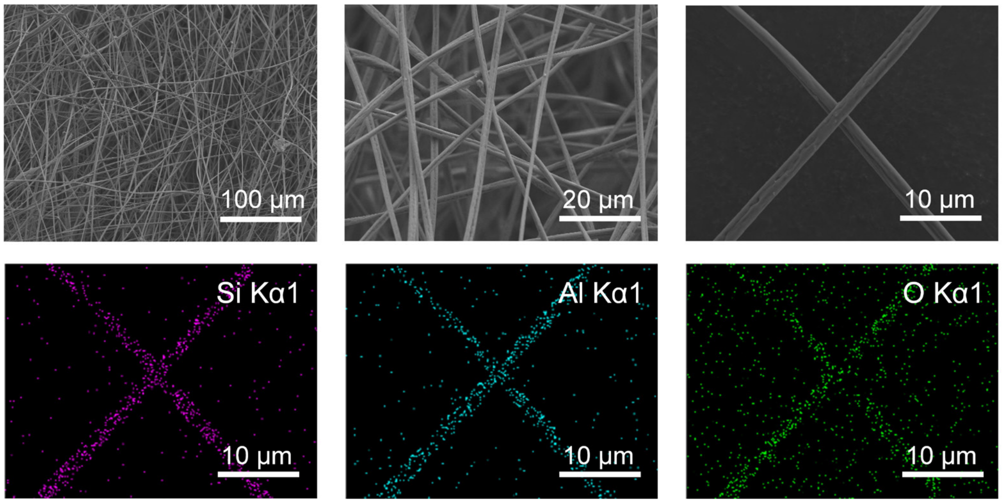

Fig. S26. SEM and EDS images of SiO_2-Al_2O_3 composite ceramic fiber mat.

3. Tables

Table S1. Comparison between KV-SBS and some other spinning methods.

Method	Spinneret	Polymer species	Average fiber diameter (nm)	Production rate (g/h)	Production rate per jet (g/h per jet)	Ref.
Needleless electrospinning (ES)	Bead structure nozzle	PAN	420	0.918	0.918	(*43*)
Multinozzle ES	Porous hollow tube	PVP	~330[1]	0.5 (20 holes)	0.025	(*44*)
Multinozzle ES	nozzles	PEO	~300[1]	0.618-0.712 (19 nozzles)	0.0325-0.0375	(*45*)
Multinozzle ES	Tubes with holes	SU-8	~200	0.75 (16 holes)	0.047	(*46*)
Needleless ES	Needle-disk	PVDF-HFP	245.23±33.75	13.5 (24 needles)	0.563	(*47*)
Multinozzle ES	Four-hole spinneret	PEO	~220-240	1.68 (4 holes)	0.42	(*48*)
Multinozzle ES	Plastic filter with holes	PEO	225	~2 (7 holes)	~0.3	(*49*)
Needleless ES	Bead wire	PVP	200-650	40 (200 beads)	0.2	(*50*)
Convex needle ES	Convex needle	PAN	~200[1]	0.3	0.3	(*51*)
Needleless ES	Tube with an embedded wire loop	PAN	150[1]	0.48	0.48	(*52*)
ES	needle	PA612	150±40	0.29	0.29	(*53*)
Multinozzle ES	Multiple pins	PVA	160.5±48.9	1.69 (21 pins)	0.08	(*54*)
Needleless ES	Bowl	PEO	268±25	0.0329±0.0083	0.0329±0.0083	(*55*)
Needleless ES	Plate edge	PEO	290±41	0.27	0.27	(*56*)
Melt blowing		PP	5000±2000	14.796	14.796	(*16*)
Melt blowing		PP	1500	12.84	12.84	(*65*)
Centrifugal spinning		EC/PVP	2000–4000	/	/	(*66*)
Centrifugal spinning		PS	4210–6000	/	/	(*31*)
**This work**	**Nylon thread**	**PAN**	**151±32**	**0.81±0.09**	**0.81±0.09**	**This work**
			**194±49**	**1.52±0.14**	**1.52±0.14**	
			**265±68**	**2.36±0.33**	**2.36±0.33**	
			**443±111**	**4.29±0.35**	**4.29±0.35**	
			**600±170**	**5.90±0.19**	**5.90±0.19**	

1: Estimated from graph.

Table S2. Comparison between PS nanofiber sponge via KV-SBS and various sorbent materials.

Absorbent Material	Adsorbed Substances	Adsorption capacities(g/g)	Ref.
Magnetic exfoliated graphite	Oils	30–50	(*67*)
Graphene/a-FeOOH composite	Cyclohexane, toluene, vegetable oil, etc.	10–30	(*68*)
Marshmallow-like porous gels	Oils and organic solvents	6–15	(*69*)
Reduced graphite oxide foam	Motor oil and organic solvents	5–40	(*70*)
Activated carbons	Benzene and toluene	<1	(*71*)
Nanowire membrane	Oils and organic solvents	4–20	(*72*)
Porous boron nitride nanosheets	Ethanol, toluene, pump oil, used engine oil and ethylene glycol	20–33	(*73*)
Cellulose Nanofibril Aerogels	Hexane, methanol, toluene, DMF, DMSO, chloroform	25–47	(*74*)
Bacterial cellulose/silica aerogels	Oils and organic solvents	8–14	(*75*)
CR-PBz-sponge	Oils and organic solvents	55–120	(*76*)
Porous PVDF–MWCNT Foam	Motor oil, rapeseed oil, and some organic solvents	3–12	(*77*)
Polydimethylsiloxane functionalized Melamine Sponge	Oils and organic solvents	15–75	(*78*)
Hydrophobic cellulose aerogel	Pump oil and chloroform	28.56–39.59	(*79*)
Cellulose acetate butyrate nanofibers	Motor oil	60	(*80*)
Carbon nanofiber aerogels	Oils and organic solvents	215–913	(*81*)
Twisted carbon fiber (TCF) aerogel	Oils and organic solvents	50–192	(*82*)
**PS nanofiber sponge**	**Oils and organic solvents**	**33–170**	**This work**

4. Supplementary Movie Legends

Movie S1. The KV-SBS process with a single nylon thread (played at 1x speed).

Movie S2. High-speed video of the KV-SBS process with a single nylon thread (played at 1/333x speed).

Movie S3. High-speed video of the solution jet formation (played at 1/333x speed).

Movie S4. CFD simulations of KV-SBS process.

Movie S5. High-speed video of the KV-SBS process with different spinning solutions (played at 1/333x speed).

Movie S6. High-speed video of the KV-SBS process with various threads (played at 1/333x speed).

Movie S7. The KV-SBS process with multiple parallel nylon threads (played at 1x speed).

Movie S8. High-speed video of the KV-SBS process with multiple parallel nylon threads (played at 1/333x speed).

Movie S9. The KV-SBS process with a mesh (played at 1x speed).

Movie S10. High-speed video of the KV-SBS process with a mesh (played at 1/333 speed).

REFERENCES AND NOTES

1. X. Liao, M. Dulle, E. S. J. M. de Souza, R. B. Wehrspohn, S. Agarwal, S. Forster, H. Hou, P. Smith, A. Greiner, High strength in combination with high toughness in robust and sustainable polymeric materials. *Science* **366**, 1376–1379 (2019).

2. G. M. Gonzalez, J. Ward, J. Song, K. Swana, S. A. Fossey, J. L. Palmer, F. W. Zhang, V. M. Lucian, L. Cera, J. F. Zimmerman, F. J. Burpo, K. K. Parker, Para-aramid fiber sheets for simultaneous mechanical and thermal protection in extreme environments. *Matter* **3**, 742–758 (2020).

3. K. C. K. Cheng, M. A. Bedolla-Pantoja, Y. K. Kim, J. V. Gregory, F. Xie, A. de France, C. Hussal, K. Sun, N. L. Abbott, J. Lahann, Templated nanofiber synthesis via chemical vapor polymerization into liquid crystalline films. *Science* **362**, 804–808 (2018).

4. Y. Yao, Z. Huang, P. Xie, L. Wu, L. Ma, T. Li, Z. Pang, M. Jiao, Z. Liang, J. Gao, Y. He, D. J. Kline, M. R. Zachariah, C. Wang, J. Lu, T. Wu, T. Li, C. Wang, R. Shahbazian-Yassar, L. Hu, High temperature shockwave stabilized single atoms. *Nat. Nanotechnol.* **14**, 851–857 (2019).

5. M. Peydayesh, R. Mezzenga, Protein nanofibrils for next generation sustainable water purification. *Nat. Commun.* **12**, 3248 (2021).

6. W. Liu, S. W. Lee, D. Lin, F. Shi, S. Wang, A. D. Sendek, Y. Cui, Enhancing ionic conductivity in composite polymer electrolytes with well-aligned ceramic nanowires. *Nat. Energy* **2**, 17035 (2017).

7. S. S. Shinde, J. Y. Jung, N. K. Wagh, C. H. Lee, D.-H. Kim, S.-H. Kim, S. U. Lee, J.-H. Lee, Ampere-hour-scale zinc–air pouch cells. *Nat. Energy* **6**, 592–604 (2021).

8. S. Lee, D. Sasaki, D. Kim, M. Mori, T. Yokota, H. Lee, S. Park, K. Fukuda, M. Sekino, K. Matsuura, T. Shimizu, T. Someya, Ultrasoft electronics to monitor dynamically pulsing cardiomyocytes. *Nat. Nanotechnol.* **14**, 156–160 (2019).

9. Z. Ma, Q. Huang, Q. Xu, Q. Zhuang, X. Zhao, Y. Yang, H. Qiu, Z. Yang, C. Wang, Y. Chai, Z. Zheng, Permeable superelastic liquid-metal fibre mat enables biocompatible and monolithic stretchable electronics. *Nat. Mater.* **20**, 859–868 (2021).

10. M. Rahmati, D. K. Mills, A. M. Urbanska, M. R. Saeb, J. R. Venugopal, S. Ramakrishna, M. Mozafari, Electrospinning for tissue engineering applications. *Prog. Mater. Sci.* **117**, 100721 (2021).

11. Q. F. Guan, H. B. Yang, Z. M. Han, L. C. Zhou, Y. B. Zhu, Z. C. Ling, H. B. Jiang, P. F. Wang, T. Ma, H. A. Wu, S. H. Yu, Lightweight, tough, and sustainable cellulose nanofiber-derived bulk structural materials with low thermal expansion coefficient. *Sci. Adv.* **6**, eaaz1114 (2020).

12. A. Palika, A. Armanious, A. Rahimi, C. Medaglia, M. Gasbarri, S. Handschin, A. Rossi, M. O. Pohl, I. Busnadiego, C. Gübeli, R. B. Anjanappa, S. Bolisetty, M. Peydayesh, S. Stertz, B. G. Hale, C. Tapparel, F. Stellacci, R. Mezzenga, An antiviral trap made of protein nanofibrils and iron oxyhydroxide nanoparticles. *Nat. Nanotechnol.* **16**, 918–925 (2021).

13. S. Talebian, G. G. Wallace, A. Schroeder, F. Stellacci, J. Conde, Nanotechnology-based disinfectants and sensors for SARS-CoV-2. *Nat. Nanotechnol.* **15**, 618–621 (2020).

14. J. Yan, Y. Zhang, Y. Zhao, J. Song, S. Xia, S. Liu, J. Yu, B. Ding, Transformation of oxide ceramic textiles from insulation to conduction at room temperature. *Sci. Adv.* **6**, eaay8538 (2020).

15. Y. Gao, J. Zhang, Y. Su, H. Wang, X. X. Wang, L. P. Huang, M. Yu, S. Ramakrishna, Y. Z. Long, Recent progress and challenges in solution blow spinning. *Mater. Horiz.* **8**, 426–446 (2021).

16. N. Fedorova, B. Pourdeyhimi, High strength nylon micro- and nanofiber based nonwovens via spunbonding. *J. Appl. Polym. Sci.* **104**, 3434–3442 (2007).

17. R. L. Shambaugh, A macroscopic view of the melt-blowing process for producing microfibers. *Ind. Eng. Chem. Res.* **27**, 2363–2372 (1988).

18. J. Xue, T. Wu, Y. Dai, Y. Xia, Electrospinning and electrospun nanofibers: Methods, materials, and applications. *Chem. Rev.* **119**, 5298–5415 (2019).

19. A. Greiner, J. H. Wendorff, Electrospinning: A fascinating method for the preparation of ultrathin fibers. *Angew. Chem. Int. Ed.* **46**, 5670–5703 (2007).

20. S. Agarwal, A. Greiner, J. H. Wendorff, Functional materials by electrospinning of polymers. *Prog. Polym. Sci.* **38**, 963–991 (2013).

21. X. Zhang, Y. Lu, Centrifugal spinning: An alternative approach to fabricate nanofibers at high speed and low cost. *Polym. Rev.* **54**, 677–701 (2014).

22. J. J. Rogalski, C. W. M. Bastiaansen, T. Peijs, Rotary jet spinning review–A potential high yield future for polymer nanofibers. *Nanocomposites* **3**, 97–121 (2017).

23. M. Yaman, T. Khudiyev, E. Ozgur, M. Kanik, O. Aktas, E. O. Ozgur, H. Deniz, E. Korkut, M. Bayindir, Arrays of indefinitely long uniform nanowires and nanotubes. *Nat. Mater.* **10**, 494–501 (2011).

24. S. K. Smoukov, T. Tian, N. Vitchuli, S. Gangwal, P. Geisen, M. Wright, E. Shim, M. Marquez, J. Fowler, O. D. Velev, Scalable liquid shear-driven fabrication of polymer nanofibers. *Adv. Mater.* **27**, 2642–2647 (2015).

25. E. S. Medeiros, G. M. Glenn, A. P. Klamczynski, W. J. Orts, L. H. C. Mattoso, Solution blow spinning: A new method to produce micro- and nanofibers from polymer solutions. *J. Appl. Polym. Sci.* **113**, 2322–2330 (2009).

26. J. Song, Z. Li, H. Wu, Blowspinning: A new choice for nanofibers. *ACS Appl. Mater. Interfaces* **12**, 33447–33464 (2020).

27. Kenry, C. T. Lim, Nanofiber technology: Current status and emerging developments. *Prog. Polym. Sci.* **70**, 1–17 (2017).

28. Y. Polat, E. S. Pampal, E. Stojanovska, R. Simsek, A. Hassanin, A. Kilic, A. Demir, S. Yilmaz, Solution blowing of thermoplastic polyurethane nanofibers: A facile method to produce flexible porous materials. *J. Appl. Polym. Sci.* **133**, 43025 (2016).

29. J. L. Daristotle, A. M. Behrens, A. D. Sandler, P. Kofinas, A review of the fundamental principles and applications of solution blow spinning. *ACS Appl. Mater. Interfaces* **8**, 34951–34963 (2016).

30. L. Persano, A. Camposeo, C. Tekmen, D. Pisignano, Industrial upscaling of electrospinning and applications of polymer nanofibers: A review. *Macromol. Mater. Eng.* **298**, 504–520 (2013).

31. H. N. Doan, D. K. Nguyen, P. P. Vo, K. Hayashi, K. Kinashi, W. Sakai, N. Tsutsumi, D. P. Huynh, Facile and scalable fabrication of porous polystyrene fibers for oil removal by centrifugal spinning. *ACS Omega* **4**, 15992–16000 (2019).

32. Y. Huang, J. Song, C. Yang, Y. Long, H. Wu, Scalable manufacturing and applications of nanofibers. *Mater. Today* **28**, 98–113 (2019).

33. T. V. Kármán, Ueber den Mechanismus des Widerstandes, den ein bewegter Körper in einer Flüssigkeit erfährt. *Nachr. Ges. Wiss. Gottingen, Math.-Phys. Kl.* **1911**, 509–517 (1911).

34. E. Gauger, H. Stark, Numerical study of a microscopic artificial swimmer. *Phys. Rev. E* **74**, 021907 (2006).

35. G. R. Hunt, D. B. Ingham, Laminar and turbulent radial jets. *Acta Mech.* **127**, 25–38 (1998).

36. X.-k. Wang, S. K. Tan, Environmental fluid dynamics-jet flow. *J. Hydrodyn.* **22**, 962–967 (2010).

37. C. Jia, L. Li, Y. Liu, B. Fang, H. Ding, J. Song, Y. Liu, K. Xiang, S. Lin, Z. Li, W. Si, B. Li, X. Sheng, D. Wang, X. Wei, H. Wu, Highly compressible and anisotropic lamellar ceramic sponges with superior thermal insulation and acoustic absorption performances. *Nat. Commun.* **11**, 3732 (2020).

38. J. Yan, Y. Wang, Y. Zhang, S. Xia, J. Yu, B. Ding, Direct magnetic reinforcement of electrocatalytic ORR/OER with electromagnetic induction of magnetic catalysts. *Adv. Mater.* **33**, e2007525 (2021).

39. X. Peng, K. Dong, C. Ye, Y. Jiang, S. Zhai, R. Cheng, D. Liu, X. Gao, J. Wang, Z. L. Wang, A breathable, biodegradable, antibacterial, and self-powered electronic skin based on all-nanofiber triboelectric nanogenerators. *Sci. Adv.* **6**, eaba9624 (2020).

40. H. Jin, M. O. G. Nayeem, S. Lee, N. Matsuhisa, D. Inoue, T. Yokota, D. Hashizume, T. Someya, Highly durable nanofiber-reinforced elastic conductors for skin-tight electronic textiles. *ACS Nano* **13**, 7905–7912 (2019).

41. D. Wang, J. A. Song, J. Wen, Y. H. Yuan, Z. L. Liu, S. Lin, H. Y. Wang, H. L. Wang, S. L. Zhao, X. M. Zhao, M. H. Fang, M. Lei, B. Li, N. Wang, X. L. Wang, H. Wu, Significantly enhanced uranium extraction from seawater with mass produced fully amidoximated nanofiber adsorbent. *Adv. Energy Mater.* **8**, 1802607 (2018).

42. L. Persano, C. Dagdeviren, Y. Su, Y. Zhang, S. Girardo, D. Pisignano, Y. Huang, J. A. Rogers, High performance piezoelectric devices based on aligned arrays of nanofibers of poly(vinylidenefluoride-co-trifluoroethylene). *Nat. Commun.* **4**, 1633 (2013).

43. Y. Zhang, Z. Cheng, Z. Han, S. Zhao, X. Zhao, L. Kang, Stable multi-jet electrospinning with high throughput using the bead structure nozzle. *RSC Adv.* **8**, 6069–6074 (2018).

44. J. S. Varabhas, G. G. Chase, D. H. Reneker, Electrospun nanofibers from a porous hollow tube. *Polymer* **49**, 4226–4229 (2008).

45. G. Zheng, J. Jiang, D. Chen, J. Liu, Y. Liu, J. Zheng, X. Wang, W. Li, Multinozzle high efficiency electrospinning with the constraint of sheath gas. *J. Appl. Polym. Sci.* **136**, 47574 (2019).

46. S.-P. Fang, P. Jao, D. E. Senior, K.-T. Kim, Y.-K. Yoon, Study on high throughput nanomanufacturing of photopatternable nanofibers using tube nozzle electrospinning with multi-tubes and multi-nozzles. *Micro Nano Syst. Lett.* **5**, 10 (2017).

47. Z. Liu, K. K. J. Ang, J. He, Needle-disk electrospinning inspired by natural point discharge. *J. Mater. Sci.* **52**, 1823–1830 (2016).

48. F.-L. Zhou, R.-H. Gong, I. Porat, Polymeric nanofibers via flat spinneret electrospinning. *Polym. Eng. Sci.* **49**, 2475–2481 (2009).

49. A. Kumar, M. Wei, C. Barry, J. Chen, J. Mead, Controlling fiber repulsion in multijet electrospinning for higher throughput. *Macromol. Mater. Eng.* **295**, 701–708 (2010).

50. S. L. Liu, Y. Y. Huang, H. D. Zhang, B. Sun, J. C. Zhang, Y. Z. Long, Needleless electrospinning for large scale production of ultrathin polymer fibres. *Mater. Res. Innov.* **18**, S4-833-S4-837 (2014).

51. I. Jahan, A. Jadhav, L. Wang, X. Wang, Electrospinning from a convex needle with multiple jet toward better controlling and enhanced production rate. *J. Appl. Polym. Sci.* **136**, 48014 (2019).

52. I. Jahan, L. Wang, X. Wang, Needleless electrospinning from a tube with an embedded wire loop. *Macromol. Mater. Eng.* **304**, 1800588 (2019).

53. P. Heikkilä, A. Taipale, M. Lehtimäki, A. Harlin, Electrospinning of polyamides with different chain compositions for filtration application. *Polym. Eng. Sci.* **48**, 1168–1176 (2008).

54. G. T. V. Prabu, B. Dhurai, A novel profiled multi-pin electrospinning system for nanofiber production and encapsulation of nanoparticles into nanofibers. *Sci. Rep.* **10**, 4302 (2020).

55. N. M. Thoppey, J. R. Bochinski, L. I. Clarke, R. E. Gorga, Edge electrospinning for high throughput production of quality nanofibers. *Nanotechnology* **22**, 345301 (2011).

56. N. M. Thoppey, J. R. Bochinski, L. I. Clarke, R. E. Gorga, Unconfined fluid electrospun into high quality nanofibers from a plate edge. *Polymer* **51**, 4928–4936 (2010).

57. J. Eggers, Drop formation–An overview. *ZAMM* **85**, 400–410 (2005).

58. T. Massalha, R. M. Digilov, The shape function of a free-falling laminar jet: Making use of Bernoulli's equation. *Am. J. Phys.* **81**, 733–737 (2013).

59. O. Regev, S. Vandebril, E. Zussman, C. Clasen, The role of interfacial viscoelasticity in the stabilization of an electrospun jet. *Polymer* **51**, 2611–2620 (2010).

60. W.-X. Huang, C. B. Chang, H. J. Sung, An improved penalty immersed boundary method for fluid–flexible body interaction. *J. Comput. Phys.* **230**, 5061–5079 (2011).

61. W.-X. Huang, S. J. Shin, H. J. Sung, Simulation of flexible filaments in a uniform flow by the immersed boundary method. *J. Comput. Phys.* **226**, 2206–2228 (2007).

62. A. M. Roma, C. S. Peskin, M. J. Berger, An adaptive version of the immersed boundary method. *J. Comput. Phys.* **153**, 509–534 (1999).

63. K. Kim, S.-J. Baek, H. J. Sung, An implicit velocity decoupling procedure for the incompressible Navier-Stokes equations. *Int. J. Numer. Methods Fluids* **38**, 125–138 (2002).

64. S. Lardeau, É. Lamballais, J.-P. Bonnet, Direct numerical simulation of a jet controlled by fluid injection. *J. Turbul.* **3**, N2 (2002).

65. M. A. Hassan, B. Y. Yeom, A. Wilkie, B. Pourdeyhimi, S. A. Khan, Fabrication of nanofiber meltblown membranes and their filtration properties. *J. Membr. Sci.* **427**, 336–344 (2013).

66. T. Hou, X. Li, Y. Lu, B. Yang, Highly porous fibers prepared by centrifugal spinning. *Mater. Des.* **114**, 303–311 (2017).

67. G. Wang, Q. Sun, Y. Zhang, J. Fan, L. Ma, Sorption and regeneration of magnetic exfoliated graphite as a new sorbent for oil pollution. *Desalination* **263**, 183–188 (2010).

68. H. P. Cong, X. C. Ren, P. Wang, S. H. Yu, Macroscopic multifunctional graphene-based hydrogels and aerogels by a metal ion induced self-assembly process. *ACS Nano* **6**, 2693–2703 (2012).

69. G. Hayase, K. Kanamori, M. Fukuchi, H. Kaji, K. Nakanishi, Facile synthesis of marshmallow-like macroporous gels usable under harsh conditions for the separation of oil and water. *Angew. Chem. Int. Ed. Eng.* **52**, 1986–1989 (2013).

70. Z. Niu, J. Chen, H. H. Hng, J. Ma, X. Chen, A leavening strategy to prepare reduced graphene oxide foams. *Adv. Mater.* **24**, 4144–4150 (2012).

71. M. A. Lillo-Ródenas, D. Cazorla-Amorós, A. Linares-Solano, Behaviour of activated carbons with different pore size distributions and surface oxygen groups for benzene and toluene adsorption at low concentrations. *Carbon* **43**, 1758–1767 (2005).

72. J. Yuan, X. Liu, O. Akbulut, J. Hu, S. L. Suib, J. Kong, F. Stellacci, Superwetting nanowire membranes for selective absorption. *Nat. Nanotechnol.* **3**, 332–336 (2008).

73. W. Lei, D. Portehault, D. Liu, S. Qin, Y. Chen, Porous boron nitride nanosheets for effective water cleaning. *Nat. Commun.* **4**, 1777 (2013).

74. A. Mulyadi, Z. Zhang, Y. Deng, Fluorine-free oil absorbents made from cellulose nanofibril aerogels. *ACS Appl. Mater. Interfaces* **8**, 2732–2740 (2016).

75. J. He, H. Zhao, X. Li, D. Su, F. Zhang, H. Ji, R. Liu, Superelastic and superhydrophobic bacterial cellulose/silica aerogels with hierarchical cellular structure for oil absorption and recovery. *J. Hazard. Mater.* **346**, 199–207 (2018).

76. C.-T. Liu, P.-K. Su, C.-C. Hu, J.-Y. Lai, Y.-L. Liu, Surface modification of porous substrates for oil/water separation using crosslinkable polybenzoxazine as an agent. *J. Membr. Sci.* **546**, 100–109 (2018).

77. F. Chen, Y. Lu, X. Liu, J. Song, G. He, M. K. Tiwari, C. J. Carmalt, I. P. Parkin, Table salt as a template to prepare reusable porous PVDF-MWCNT foam for separation of immiscible oils/organic solvents and corrosive aqueous solutions. *Adv. Funct. Mater.* **27**, 1702926 (2017).

78. X. Chen, J. A. Weibel, S. V. Garimella, Continuous oil–water separation using polydimethylsiloxane-functionalized melamine sponge. *Ind. Eng. Chem. Res.* **55**, 3596–3602 (2016).

79. A. Sanguanwong, P. Pavasant, T. Jarunglumlert, K. Nakagawa, A. Flood, C. Prommuak, Hydrophobic cellulose aerogel from waste napkin paper for oil sorption applications. *Nord. Pulp Pap. Res. J.* **35**, 137–147 (2020).

80. A. Tanvir, V. P. Ting, S. J. Eichhorn, Nanoporous electrospun cellulose acetate butyrate nanofibres for oil sorption. *Mater. Lett.* **261**, 127116 (2020).

81. Z. Y. Wu, C. Li, H. W. Liang, J. F. Chen, S. H. Yu, Ultralight, flexible, and fire-resistant carbon nanofiber aerogels from bacterial cellulose. *Angew. Chem. Int. Ed. Eng.* **52**, 2925–2929 (2013).

82. H. Bi, Z. Yin, X. Cao, X. Xie, C. Tan, X. Huang, B. Chen, F. Chen, Q. Yang, X. Bu, X. Lu, L. Sun, H. Zhang, Carbon fiber aerogel made from raw cotton: A novel, efficient and recyclable sorbent for oils and organic solvents. *Adv. Mater.* **25**, 5916–5921 (2013).
